# A Nuclear Hormone Receptor *nhr‐76* Induces Age‐Dependent Chemotaxis Decline in *C. elegans*

**DOI:** 10.1111/acel.70277

**Published:** 2025-10-23

**Authors:** Rikuou Yokosawa, Kentaro Noma

**Affiliations:** ^1^ Graduate School of Science Nagoya University Nagoya Japan

**Keywords:** behavioral aging, *C. elegans*, genetic program, nuclear hormone receptor, olfaction

## Abstract

A decline in food‐searching behavior of post‐reproductive animals can benefit the population and possibly be programmed by the genome despite its detrimental effect on an individual. We investigated the genetic program of age‐dependent decline in chemotaxis behavior toward an odorant secreted from bacterial food in *C. elegans* . Through a novel forward genetic screen, we identified the gene encoding a nuclear hormone receptor, *nhr‐76*, whose mutants ameliorate the age‐dependent chemotaxis decline. We found that NHR‐76 downregulates odorant receptor expression during aging in a ligand‐binding‐domain–dependent manner. Since NHR‐76 expression and localization remain unchanged with age, its activity may be modulated through the ligand‐binding domain, leading to age‐dependent chemotaxis decline. Our findings imply that post‐reproductive behavioral decline can be genetically programmed.

## Graphical abstract

A forward genetic screen in *C. elegans* identified a nuclear hormone receptor, *nhr‐76,* as a gene that programs age‐dependent behavioral decline. In post‐reproductive adults, NHR‐76 downregulates *odr‐10* that encodes an odorant receptor, leading to impaired chemotaxis.

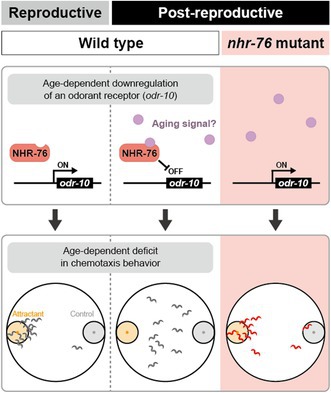

## 1 Introduction

Aging can be defined as the functional decline of an organism after sexual maturation. The aging of the nervous system leads to behavioral decline in various animals. In humans, brain aging is often associated with neurodegenerative diseases such as Alzheimer's and Parkinson's diseases, and their cellular and molecular mechanisms are extensively studied (Balestrino and Schapira 2020; Breijyeh and Karaman 2020). However, age‐related diseases do not explain all the age‐related physiological changes. So, what are the causes of neural aging? Twelve hallmarks of aging are proposed based on age‐associated manifestations and their effect on aging processes (Lopez‐Otin et al. 2023). Among these hallmarks, passive accumulation of cellular and molecular damages, such as cellular senescence, genomic instability, and loss of proteostasis, is widely accepted to cause aging (Lopez‐Otin et al. 2023). On the other hand, genetic programs can also contribute to aging, as revealed by lifespan studies. For example, a mutation in *daf‐2*, which encodes an insulin/IGF receptor, was found to double the lifespan of *C. elegans* (Kenyon et al. 1993). Further studies establish that lifespan is genetically regulated (Kenyon 2010). Compared with progress in understanding organismal lifespan, the genetic program of neuronal aging and consequent behavioral aging is not well explored. For studying the genetics of behavioral aging, *C. elegans* is ideal due to its short lifespan and robust behaviors and a simple, well‐defined nervous system (Bargmann 2006; Tissenbaum 2015). *C. elegans* experiences an age‐dependent decline in various behaviors, such as chemotaxis toward volatile odorants and associative learning behaviors (Collins et al. 2008; Stein and Murphy 2012; Suryawinata et al. 2024). At the neuronal level, behavioral aging is attributed to decreased activity of secondary olfactory neurons (Leinwand et al. 2015) or neuronal hyperactivity interfering with the circuit (Aleogho et al. 2025); whole‐brain imaging has revealed the global imbalance of brain activity (Wirak et al. 2022). At the molecular level, the mutation of a gene encoding kynurenic acid metabolism or the activation of a G protein alpha subunit can ameliorate butanone‐associative learning behavior (Arey et al. 2018; Vohra et al. 2018). However, the active genetic program that causes behavioral aging is still largely unknown.

Forward genetic screening in *C. elegans* has driven unexpected discoveries and a comprehensive understanding of the genetic program of various biological processes (Singh 2021). The lack of understanding of the active genetic program underlying behavioral aging can be partly due to the limited application of forward genetic screening to behavioral aging. In this study, we aimed to establish a novel forward genetic screen based on behavioral aging. We focused on chemotaxis behavior toward diacetyl in *C. elegans* because it is well characterized in young animals (Bargmann et al. 1993) and shows robust age‐dependent decline after the end of reproduction (Suryawinata et al. 2024). Diacetyl is derived from bacterial food (Choi et al. 2016) and sensed by the G protein‐coupled receptor, ODR‐10, specifically expressed in the AWA sensory neurons (Bargmann et al. 1993; Sengupta et al. 1996). The expression of ODR‐10 declined during aging, consistent with chemotaxis decline (Suryawinata et al. 2024).

In this study, a forward genetic screen identified a loss‐of‐function mutant of *nhr‐76* that does not show an age‐dependent decline in the chemotaxis behavior toward diacetyl. This discovery suggests that *nhr‐76* actively causes chemotaxis decline. *nhr‐76* encodes a nuclear hormone receptor and regulates the ODR‐10 expression age‐dependently. We propose that an active genetic mechanism underlies the defective food‐searching behavior of post‐reproductive individuals, a trait that could enhance population fitness and is therefore likely to be evolutionarily conserved.

## 2 Results

### 2.1 Forward Genetic Screen Identified the *knj39* Mutant That Ameliorated Age‐Dependent Chemotaxis Decline

**FIGURE 1:**
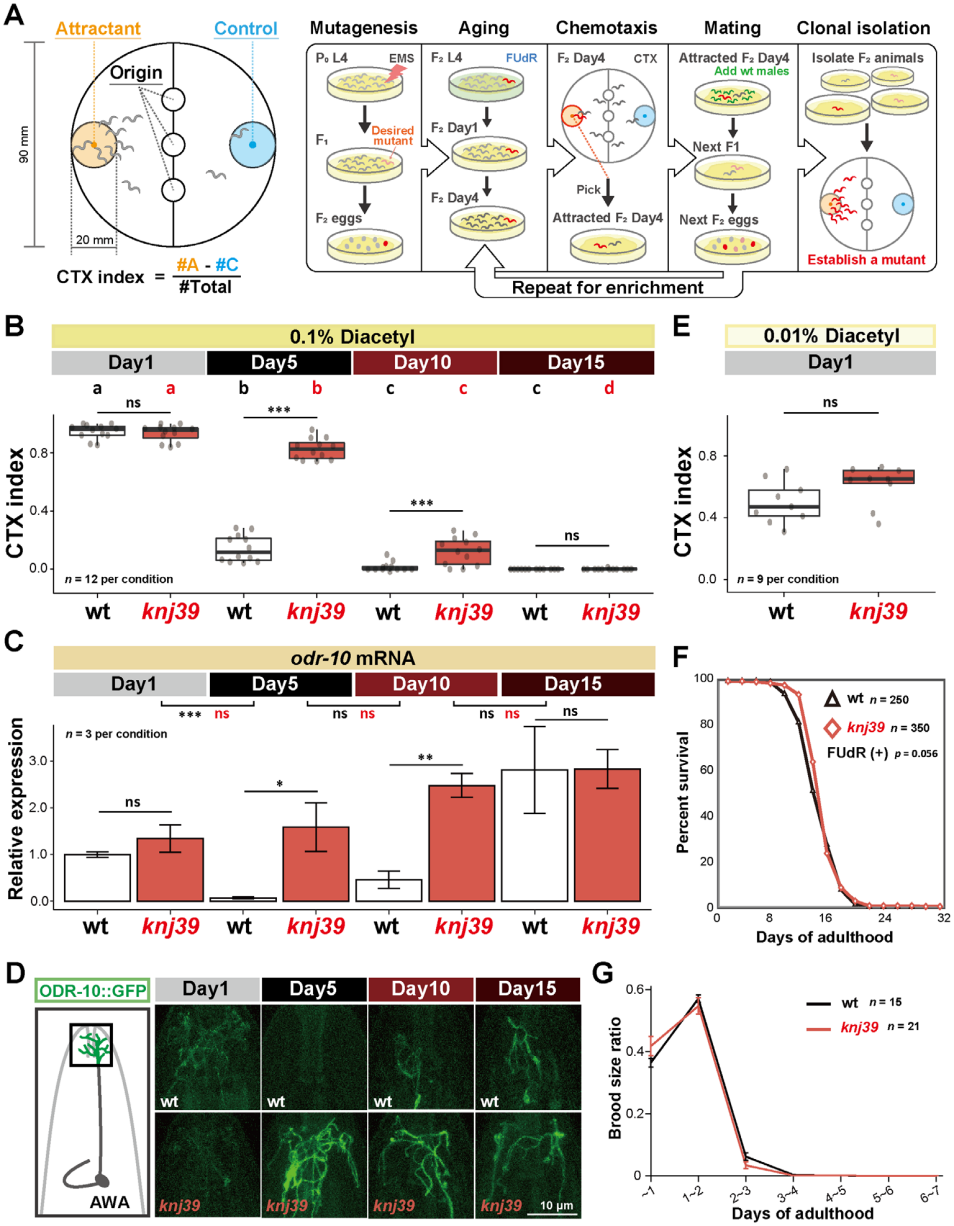
Forward genetic screen identified a mutant that ameliorates age‐dependent chemotaxis decline. (A) Schematic of population chemotaxis assay and the forward genetic screen to isolate mutants ameliorating age‐dependent chemotaxis decline. Animals' ability to chemotax toward an attractive odorant was examined. The chemotaxis (CTX) index was calculated using the indicated formula, where #A and #C indicate the number of animals in the attractant and control circles, respectively. The steps from “Aging” to “Mating” were repeated multiple times to enrich for desired mutants. In (B), (C), and (D), Day 1, Day 5, Day 10, and Day 15 animals were tested in the wild type and *knj39* mutants. (B) The quantification of chemotaxis ability toward 0.1% diacetyl. (C) The *odr‐10* mRNA expression normalized to the mean value of the Day 1 wild type. Error bars indicate SEM. (D) Schematic of the head of an animal and representative images of the ODR‐10::GFP reporter (*kyIs53[odr‐10::GFP]*), expressed in AWA sensory neurons. Scale bar = 10 μm. Gamma value = 0.64. (E) The quantification of chemotaxis ability toward 0.01% diacetyl, which is around EC50 value for wild‐type Day 1 animals. Day 1 animals of the wild type and *knj39* mutants were tested. (F) Survival curve of the wild type and *knj39* mutants with FUdR. (G) The reproductive span of the wild type and *knj39* mutants. The number of progeny deposited during the indicated period was normalized to the total number of progeny of each animal. Statistical tests were conducted using two‐way ANOVA with Tukey's test for (B). The main effects of genotype (wt vs. *knj39*), day, and their interaction were significant. Different letters indicate significant pairwise differences within the same genotype (*p* < 0.05); Student's *t*‐test between the same day groups for (C). Comparisons between different ages are indicated as black and red for *wt* and *knj39*, respectively; Mann–Whitney *U* test for (E); Log‐rank test for (F). ns: *p* > 0.05; *p < 0.05; **p < 0.01; ***p < 0.001.

The chemotaxis ability toward diacetyl declined from Day 1 of adulthood (Day 1) to Day 5 after the end of self‐reproduction of a hermaphrodite (Figure 1A, left panel, and Figure 1B, wt Day 1 and wt Day 5) (Suryawinata et al. 2024). Consistent with the behavior, the expression of the transcript of the diacetyl receptor *odr‐10* and GFP‐tagged ODR‐10 proteins declined from Day 1 to Day 5 (Figure 1C,D, and Figure S1, wt Day 1 and wt Day 5) (Suryawinata et al. 2024). On the other hand, these expressions showed a trend to increase from Day 5 to Day 10 when most animals were still alive, although the chemotaxis ability remained low on Day 10 (Figure 1B–D, Figure S1, wt). Locomotion speed continuously declined from Day 1 to Day 15 (Figure S2, wt). Since the transgenic overexpression of *odr‐10* could ameliorate the chemotaxis decline at Day 5 (Suryawinata et al. 2024), the chemotaxis decline is not solely attributable to locomotion deficits. We speculated that an active genetic mechanism might decrease the chemotaxis ability around Day 5 by decreasing *odr‐10* expression. To isolate mutants regulating age‐dependent chemotaxis decline, we sought to perform forward genetic screening. However, the mass screening with Day 5 hermaphrodites was not feasible because they did not have enough self‐fertilized progeny (Figure S3, −FUdR, male (−)). Since sperm depletion terminates self‐reproduction in *C. elegans* (Ward and Carrel 1979), we mated Day 3 hermaphrodites with Day 1 males and could obtain progeny even from Day 3 animals (Figure S3, Day 3, −FUdR, male (+); see Section 4 for details). Furthermore, we found that the transient treatment of the DNA synthesis inhibitor, FUdR (Cohen et al. 1958), further increased the progeny of Day 4 animals with male addition (Figure S3, Day 3 and Day 4, +FUdR, male (+)). Thus, we performed a forward genetic screen based on the chemotaxis ability of the F_2_ progeny of mutagenized animals at Day 4 and recovered a desired mutant's progeny by transient FUdR treatment and mating with unmutagenized Day 1 males (Figure 1A). A few rounds of mating and behavioral assays decreased false positives and enriched potential mutants (Figure 1A; see Section 4 for details).

From the screen, we isolated a mutant *knj39* that ameliorated the chemotaxis decline toward 0.1% diacetyl even at Day 5 (Figure 1B, *knj39*). Consistent with the chemotaxis behavior, the expression of *odr‐10* transcripts and GFP‐tagged ODR‐10 did not decline from Day 1 to Day 5 in the *knj39* mutants (Figure 1C,D and Figure S1, *knj39*). Despite high *odr‐10* expression, chemotaxis ability was not maintained at Day 10 (Figure 1B,C, *knj39*). The reduced chemotaxis ability at this stage might result from locomotion defects (Figure S2) and/or impaired integration of sensory information, rather than diminished sensory function. The better chemotaxis of *knj39* mutants at Day 5 was not due to the secondary effects of the better chemotaxis ability of Day 1 animals because Day 1 *knj39* mutants showed normal chemotaxis toward a lower concentration of diacetyl (Figure 1E, 0.01% diacetyl). Moreover, the lifespan of *knj39* mutants was comparable to that of the wild type in the FUdR‐treated condition (Figure 1F). In the no‐FUdR condition, *knj39* had a slightly shorter lifespan (Figure S4). Since the timing of the chemotaxis decline is correlated with the end of the self‐reproduction (Suryawinata et al. 2024), we examined the time course of the brood size of *knj39* mutants. *knj39* mutants had a similar self‐reproductive span to the wild type, with a slightly decreased total brood size (Figures 1G and Figure S5A). These results suggest that the ameliorative effect of *knj39* on the chemotaxis decline was not due to a longer lifespan or longer self‐reproductive span.

Day 1 wild‐type animals require the diacetyl receptor, *odr‐10*, for the chemotaxis behavior (Figure S6, Day 1 wt) (Sengupta et al. 1996). Similarly, Day 5 *knj39* mutants required *odr‐10* for the chemotaxis behavior (Figure S6, Day 5 *knj39*), suggesting that Day 5 *knj39* mutants did not recruit other receptors to achieve high chemotaxis toward diacetyl. Collectively, our results suggest that *knj39* mutants lack a genetic program to decrease *odr‐10* expression and chemotaxis behavior in Day 5 animals.

### 2.2 NHR‐76 Functions in AWA Neurons to Cause Age‐Dependent Chemotaxis Decline

**FIGURE 2:**
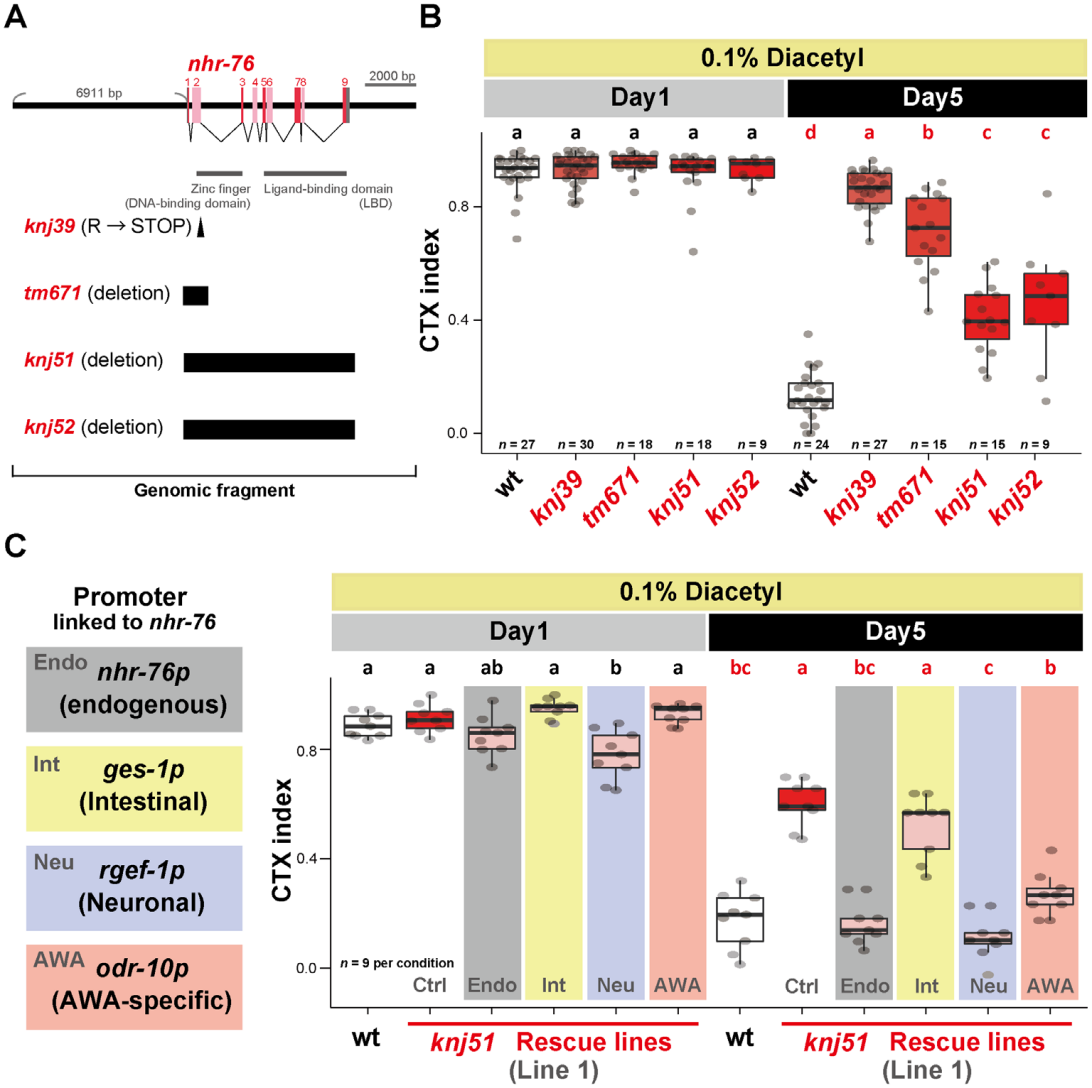
*nhr‐76* functions in AWA neurons. (A) Schematic of gene structure and alleles of *nhr‐76*. Red and pink boxes, exons; Gray boxes, 5′UTR and 3′UTR; black triangle, point mutation; black box, deletion. The exon numbers are shown above the red boxes. The genomic fragment used for rescue experiments in (C), Figure S9 and Figure S10A is indicated. In (B) and (C), synchronized Day 1 and Day 5 animals were tested for each genotype. (B) The quantification of chemotaxis ability toward 0.1% diacetyl of the wild type and mutants of four alleles: *knj39*, *tm671*, *knj51*, and *knj52*. (C) The quantification of chemotaxis ability toward 0.1% diacetyl. The wild type, *nhr‐76* (*knj51*) mutants, and the *nhr‐76* tissue/cell‐specific rescue lines were tested (Line 1, a different set of transgenic lines was shown in Figure S10A). The control strain (Ctrl) carries only the co‐injection markers (HygR and coelomocyte‐RFP). All the transgenic strains were treated with hygromycin from eggs. The PCR fragment of the *nhr‐76* genomic locus, including the *nhr‐76* endogenous promoter and 3′UTR, was used as *nhr‐76*p rescue. Intestinal (*ges‐1*p), pan‐neuronal (*rgef‐1*p), and AWA‐specific (*odr‐10*p) promoters linked to *nhr‐76* cDNA were used for tissue/cell‐specific rescue experiments. Statistical tests were conducted using two‐way ANOVA with Tukey's test. Different letters indicate significant pairwise differences within the same age group (*p* < 0.05).

Based on the chromosome linkage analysis and whole‐genome sequencing, *knj39* was predicted to be a mutation of *nhr‐76* encoding a nuclear hormone receptor (Figure 2A). The *knj39* mutant contains a substitution mutation from an arginine to a stop codon in *nhr‐76* (Figure 2A). To address whether *nhr‐76* is the causative gene of *knj39*, we examined three deletion alleles of *nhr‐76* (Figure 2A,B, *tm671*, *knj51*, and *knj52*). Mutants of the complete deletion alleles, *knj51* and *knj52*, had no detectable *nhr‐76* transcripts using RT‐qPCR (Figure S7A–C) and phenocopied *knj39* mutants in the chemotaxis assay (Figure 2B). For *tm671*, we detected *nhr‐76* transcripts and confirmed by RT‐PCR that exons 3–9 remained expressed (Figure S7A,D,E). Since *tm671* deletes the DNA‐binding domain (Figure 2A) and phenocopied the deletion alleles (Figure 2B), *tm671* appears to be a loss‐of‐function allele. The ameliorative effects of the complete deletion mutants (*knj51* and *knj52*) were weaker than those of *knj39* and *tm671*, which could be due to the background mutations in *knj39* and *tm671* (Figure 2B). It is also possible that an alternative ATG in exon 3 in the *knj39* and *tm671* mutants generated the ligand‐binding domain without the DNA‐binding domain, which somehow caused a stronger phenotype. *knj39* and *tm671* exhibited semi‐dominant phenotypes as heterozygotes, although this was not significant for *tm671* (Figure S8A,B). Since the null allele *knj51* was also semidominant (Figure S8C), *nhr‐76* likely exhibits haploinsufficiency. We confirmed that the expression of the PCR products of the *nhr‐76* genomic locus, including the 6911‐bp upstream region and the 3′UTR, rescued the chemotaxis phenotype of *nhr‐76(knj39)* and *nhr‐76(knj51)* (Figure 2C, Figures S9 and S10A, *nhr‐76*p). These results suggest that the loss of function of *nhr‐76* ameliorated age‐dependent chemotaxis decline. In contrast to the *knj39* mutants (Figure S5A), the deletion mutant, *knj51*, had a normal brood size (Figure S5B) and reproductive span (Figure S5C), excluding the pleiotropic effect of *nhr‐76* on reproduction.

Next, we sought to determine in which tissues/cells *nhr‐76* regulates *odr‐10* expression age‐dependently. For the tissue–/cell‐specific rescue experiments, we used a complete deletion allele *nhr‐76(knj51)*, instead of *knj39*. A previous study showed that *nhr‐76* regulates lipid metabolism in the intestine (Noble et al. 2013). However, intestine‐specific expression of *nhr‐76* did not rescue the *nhr‐76(knj51)* mutants' phenotype in the age‐dependent chemotaxis decline (Figure 2C, *ges‐1*p). To further examine the possible involvement of the *nhr‐76* function in the intestine, we focused on the genes in the *nhr‐76*‐related lipid metabolism pathway (Noble et al. 2013) (*ser‐6*, *tbh‐1*, *mod‐1*, and *tph‐1*). However, the mutants of these genes showed no phenotype of age‐dependent chemotaxis decline (Figure S11). These results suggest that *nhr‐76* ameliorates the age‐dependent chemotaxis decline independently of the regulation of lipid metabolism in the intestine. The *C. elegans* Neuronal Gene Expression Map & Network (CeNGEN) Project showed that *nhr‐76* is expressed in 18 neurons, including the AWA sensory neurons, where *odr‐10* is expressed specifically (Hammarlund et al. 2018). In contrast to intestinal expression, the expression of *nhr‐76* in all neurons or only in AWA sensory neurons rescued *nhr‐76(knj51)* deletion mutants' phenotype (Figure 2C, *rgef‐1*p and *odr‐10*p for pan‐neuronal and AWA‐specific expression, respectively). We further confirmed the AWA neurons as the site of action using a different AWA‐specific promoter, *odr‐7*p (Figure S10B). For all tissue‐specific rescue experiments, the same results were recapitulated with an independent set of transgenic lines (Figure S10A). Thus, *nhr‐76* regulates age‐dependent chemotaxis decline in AWA sensory neurons, where the diacetyl receptor *odr‐10* is specifically expressed (Sengupta et al. 1996).

### 2.3 NHR‐76 Inhibits ODR‐7 Age‐Dependently to Decrease the *odr‐10* Expression

**FIGURE 3:**
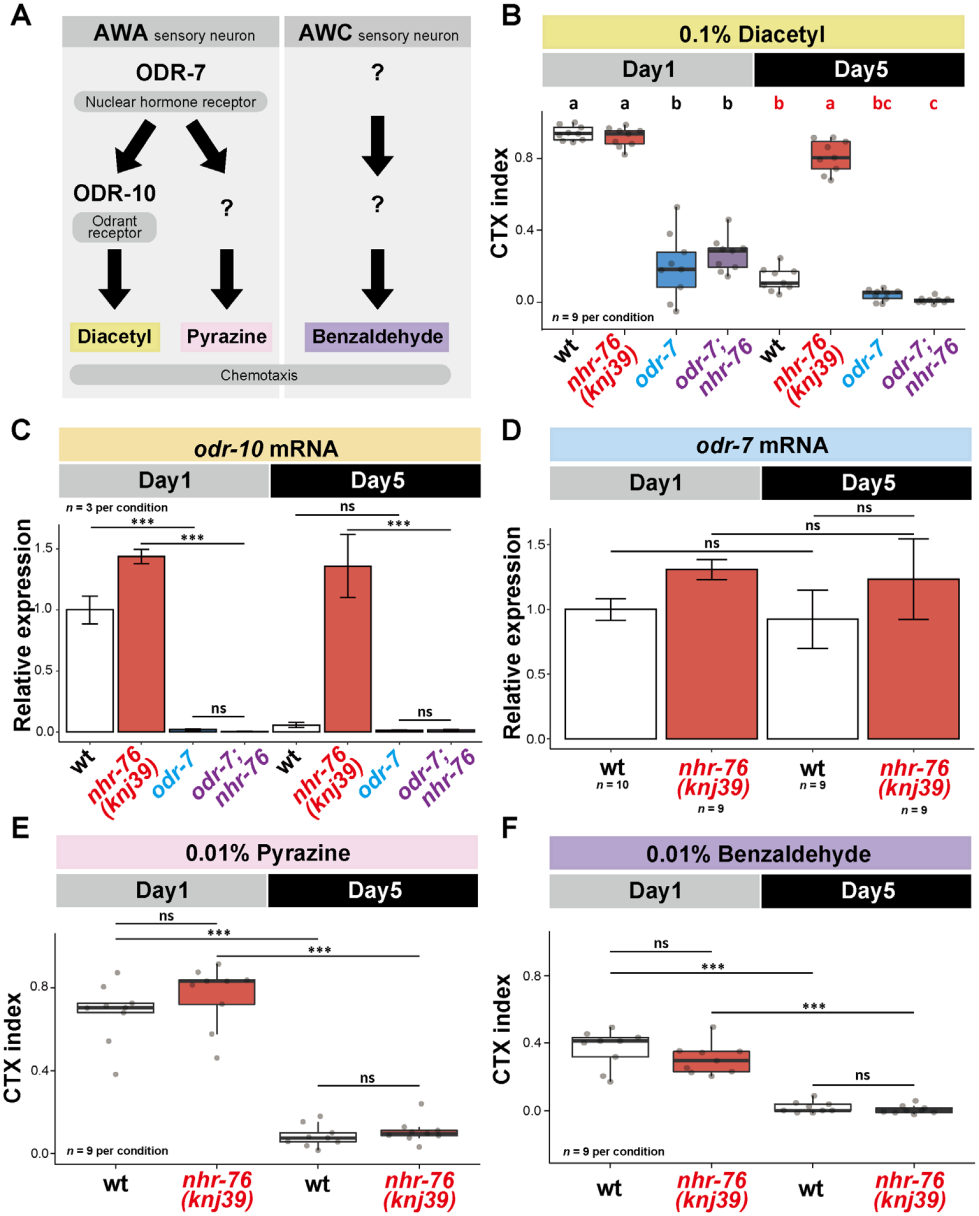
*nhr‐76* regulates chemotaxis toward diacetyl through *odr‐7*. (A) Schematic of the regulation for chemotaxis behavior toward three different attractive odorants: diacetyl, pyrazine, and benzaldehyde. The *odr‐7* gene, encoding a nuclear hormone receptor, functions in AWA neurons and is required for chemotaxis toward diacetyl and pyrazine at Day 1. *odr‐10*, encoding a GPCR, is required for chemotaxis toward diacetyl but not for pyrazine (Colosimo et al. 2003; Sengupta et al. 1996, 1994). In (B)–(F), Day 1 and Day 5 animals were tested in each genotype. (B) and (C) Epistasis analysis between *odr‐7* and *nhr‐76*. (B) The quantification of chemotaxis ability toward 0.1% diacetyl. (C) The *odr‐10* mRNA expression, normalized by the mean value of the Day 1 wild type. Error bars indicate SEM. (D) *odr‐7* mRNA expression in the wild type and *nhr‐76* mutants, normalized by the mean value of the Day 1 wild type. Error bars indicate SEM. (E) and (F) The effect of *nhr‐76* mutants on the chemotaxis ability toward 0.01% pyrazine (E) and 0.01% benzaldehyde (F). Statistical tests were conducted using two‐way ANOVA with Tukey's test for (B), (D), (E), and (F); one‐way ANOVA with Tukey's test for (C). ns: *p* > 0.05; **p < 0.01; ***p < 0.001. Different letters indicate significant pairwise differences within the same age group (*p* < 0.05).

In Day 1 animals, *odr‐7* encoding another nuclear hormone receptor is required for chemotaxis toward diacetyl by promoting the *odr‐10* expression (Colosimo et al. 2003; Sengupta et al. 1994) (Figure 3A–C, *odr‐7*). To investigate how NHR‐76 regulates the *odr‐10* expression, we analyzed the epistasis between the two nuclear hormone receptor genes, *nhr‐76* and *odr‐7*. Like Day 1 wildtype animals, *odr‐7* was required for chemotaxis behavior and the *odr‐10* expression of the Day5 *nhr‐76* mutants (Figure 3B,C). Thus, NHR‐76 regulates *odr‐10* expression through *odr‐7*. Since the expression of *odr‐7* transcripts did not drastically change during aging in the wild type and *nhr‐76* mutants (Figure 3D) (Suryawinata et al. 2024), NHR‐76 might inhibit the *odr‐7*–dependent activation of *odr‐10* at Day 5.

Next, we addressed the odorant specificity of the chemotaxis regulation by NHR‐76 using two additional odorants, pyrazine and benzaldehyde. Pyrazine is an odorant sensed by AWA neurons like diacetyl (Bargmann et al. 1993); *odr‐7*, but not *odr‐10*, is required for pyrazine chemotaxis (Sengupta et al. 1994) (Figure 3A). Benzaldehyde is sensed by another pair of sensory neurons, AWC (Bargmann et al. 1993); neither *odr‐7* nor *odr‐10* is required for benzaldehyde chemotaxis (Sengupta et al. 1996, 1994) (Figure 3A). Wild‐type animals showed a chemotaxis decline toward pyrazine and benzaldehyde (Figure 3E,F, wt). In contrast to the diacetyl chemotaxis, *nhr‐76* mutants did not ameliorate the age‐dependent chemotaxis decline toward pyrazine or benzaldehyde (Figure 3E,F, *nhr‐76*). These results suggest that NHR‐76 specifically inhibits the *odr‐10* expression to affect diacetyl chemotaxis.

### 2.4 NHR‐76 May Change Its Activity Age‐Dependently Through the Ligand‐Binding Domain

**FIGURE 4:**
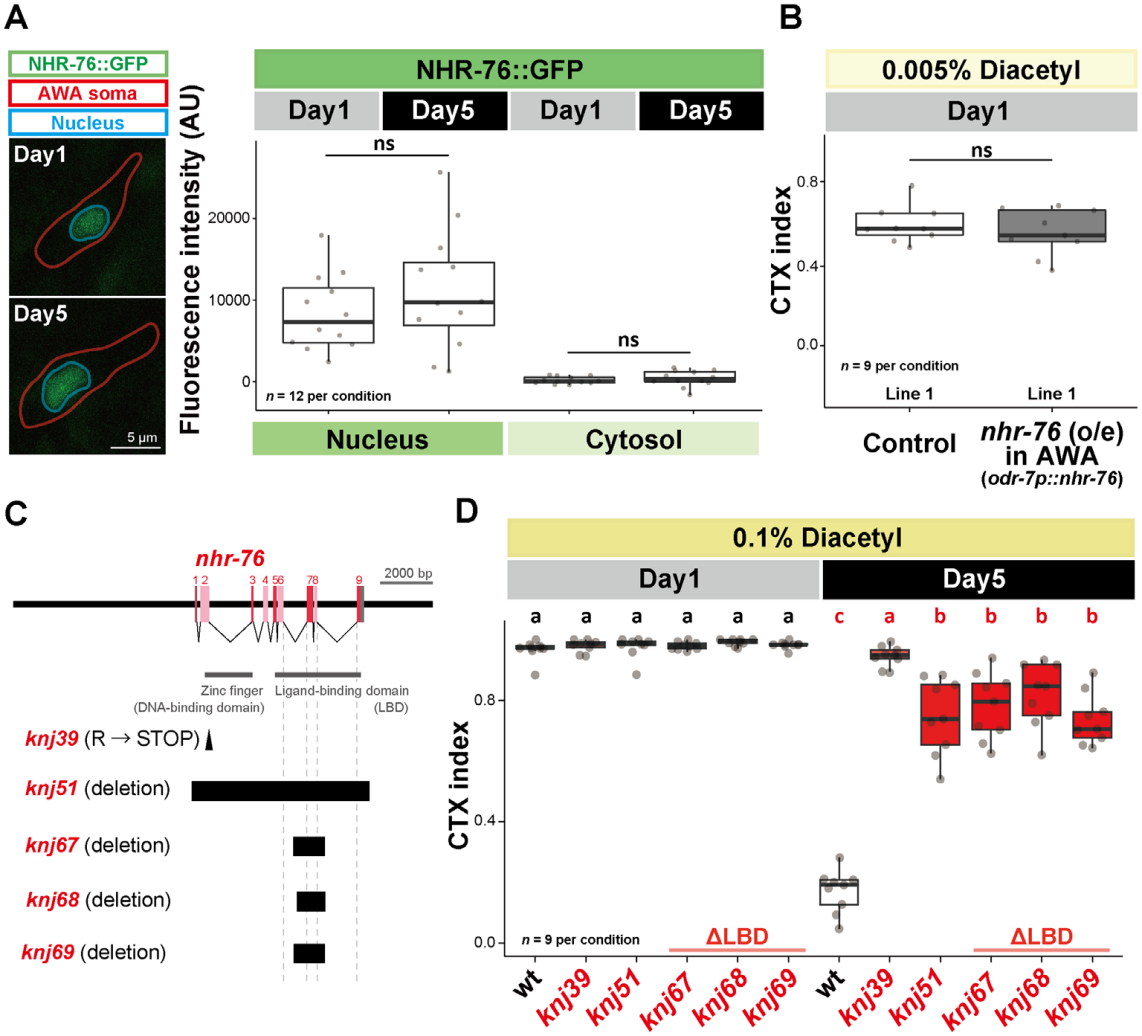
*nhr‐76* may be activated through a ligand‐binding domain. (A) The fluorescence intensity of NHR‐76::GFP reporter (*wgIs203[nhr‐76::TY1::EGFP::3xFLAG + unc‐119(+)]*) in the AWA sensory neurons. AWA sensory neurons were identified by the expression of *odr‐10p::tagRFP*. The red and blue lines indicate the AWA cell body and the nucleus, respectively. (B) The quantification of chemotaxis toward 0.005% diacetyl, which is around the EC50 for wild‐type Day 1 animals. Day 1 animals of the wild type and a transgenic strain overexpressing *nhr‐76* in AWA sensory neurons were tested. (C) Schematics of the gene structure and alleles of *nhr‐76*. Red and pink boxes, exons; Gray boxes, 5′UTR and 3′UTR; black triangle, point mutation; black box, deletion. The exon numbers were shown above the red boxes. (D) The quantification of chemotaxis toward 0.1% diacetyl. Day 1 and Day 5 animals were tested in the wild type, the *nhr‐76* loss‐of‐function mutants (*knj39* and *knj51*), and the *nhr‐76* LBD‐deleted mutants (*knj67*, *knj68*, and *knj69*). Statistical tests were conducted using Student's *t*‐test for (A); Mann–Whitney *U* test for (B); and two‐way ANOVA with Tukey's test for (D). ns: *p* > 0.05. Different letters indicate significant pairwise differences within the same age group (*p* < 0.05).

Some nuclear hormone receptors are known to translocate from the cytosol to the nucleus when activated (Aranda and Pascual 2001). To address how NHR‐76 can inhibit the *odr‐10* expression age‐dependently, we observed the GFP‐tagged NHR‐76 (NHR‐76::GFP) signals. NHR‐76::GFP expression was observed in the intestine and neurons of animals carrying a multi‐copy integrated transgene (*wgIs203[nhr‐76::TY1::EGFP::3xFLAG + unc‐119(+)]*), as previously reported (Hammarlund et al. 2018; Noble et al. 2013; Sarov et al. 2006). We focused on the AWA sensory neuron as the site of *nhr‐76* action in the age‐dependent chemotaxis decline. NHR‐76::GFP was localized primarily in the nucleus in Day 1 animals (Figure 4A, Day 1). The quantitative analysis revealed that the expression and nuclear localization of NHR‐76 did not change from Day 1 to Day 5 (Figure 4A), suggesting that the abundance or nuclear localization does not regulate NHR‐76 function. To confirm that *nhr‐76* abundance does not affect chemotaxis, we overexpressed *nhr‐76* in Day 1 animals and found that *nhr‐76* overexpression in AWA neurons did not decrease chemotaxis ability toward diacetyl at Day 1 (Figure 4B and Figure S12A,B). These results imply that NHR‐76 might require a ligand specifically expressed around Day 5 to inhibit the *odr‐10* expression. Exons 6–9 encode the ligand‐binding domain (LBD) of NHR‐76 (Figure 4C). To further investigate the requirement of ligand binding, we generated three independent *nhr‐76* mutants (*knj67‐knj69*) that have in‐frame deletions of exons 7 and 8 (Figure 4C). Sanger sequencing of the cDNA and quantitative PCR confirmed that the LBD‐deletion mutants did not have mis‐splicing or reduced expression (Figures S7B,C and S13A–E). Nonetheless, the LBD‐deletion mutants of *nhr‐76* ameliorated the age‐dependent chemotaxis decline, suggesting that the LBD of NHR‐76 is required for decreasing chemotaxis ability in an age‐dependent manner (Figure 4D).

### 2.5 NHR‐76 Regulates the Age‐Dependent Decline of Chemotaxis Toward Diacetyl‐Producing Bacteria

**FIGURE 5:**
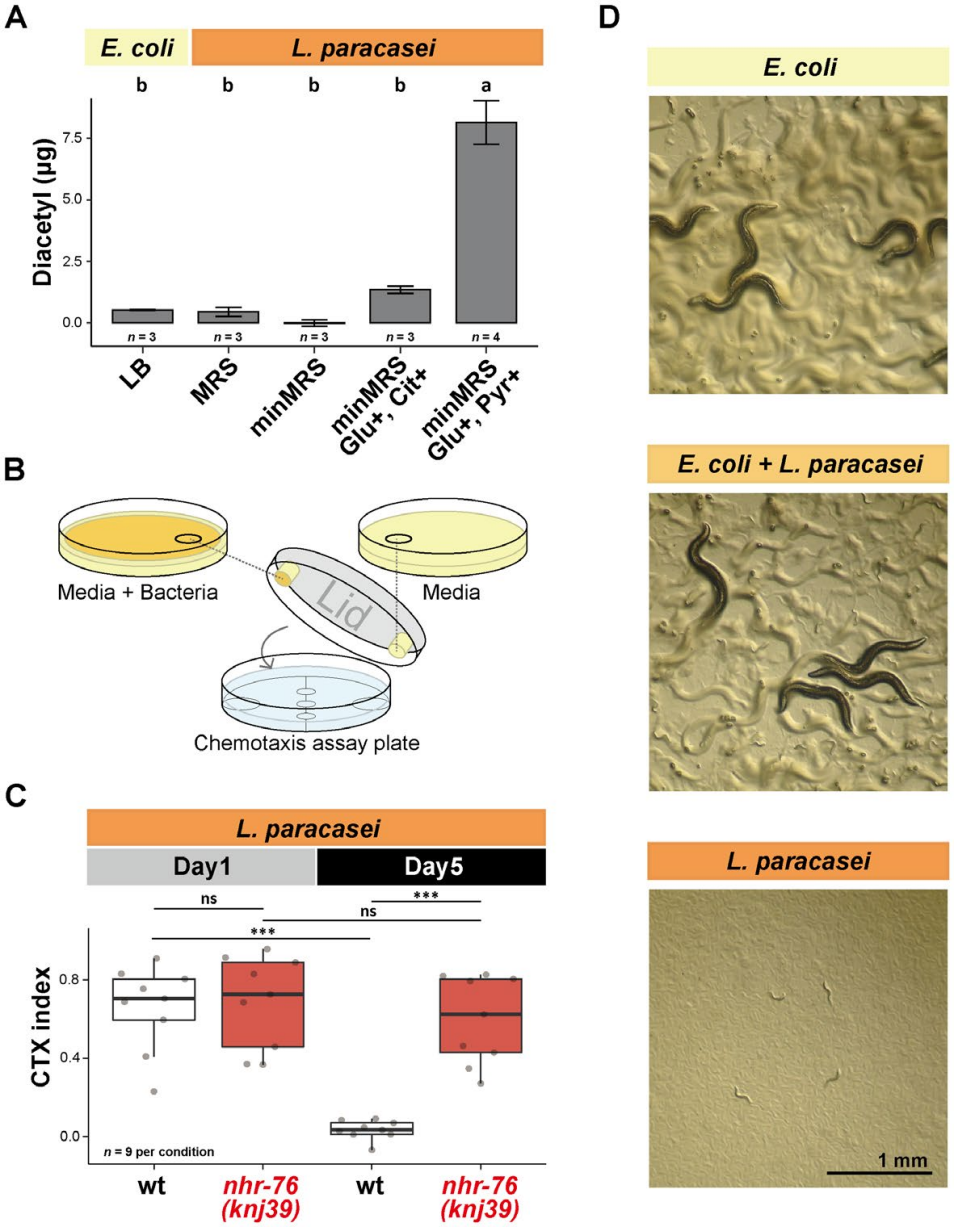
*nhr‐76* mutants ameliorate age‐dependent chemotaxis decline toward diacetyl‐producing bacteria. (A) The amount of diacetyl produced by *E. coli* or *L. paracasei* . Error bars indicate SEM. LB agar was used for *E. coli* . MRS agar, minimal MRS (minMRS) agar, minMRS agar supplemented with glucose and citrate (Glu+, Cit+), or minMRS agar supplemented with glucose and pyruvate (Glu+, Pyr+) were used for *L. paracasei* . (B) A schematic of the chemotaxis assay toward bacterial odorant. The agar plugs with or without *L. paracasei* were placed on the lid of an assay plate and not in direct contact with the assayed animals. (C) The quantification of chemotaxis ability toward *L. paracasei* cultured on minMRS supplemented with glucose and pyruvate. Day 1 and Day 5 animals were tested for the wild type and *nhr‐76 (knj39)* mutants. (D) Representative images showing the animals cultured at 23°C for 3 days from eggs with *E. coli*, the mixture of *E. coli* and *L. paracasei* , or *L. paracasei*. The stage of animals cultured on *E. coli* is considered Day 1. Scale bar: 1 mm. Statistical tests were conducted using one‐way ANOVA with Tukey's test for (A); two‐way ANOVA with Tukey's test for (C). Different letters indicate significant pairwise differences (*p* < 0.05). ns: *p* > 0.05; ***p < 0.01.

Our results so far indicate that *nhr‐76* actively causes the chemotaxis decline toward diacetyl in Day 5 animals. What is the potential benefit of this genetic program that is apparently detrimental to an individual? Diacetyl is thought to be an odorant of bacterial food for *C. elegans* (Bargmann et al. 1993). Thus, we speculated that *nhr‐76* regulates the attraction toward bacterial food in an age‐dependent manner. To address this issue, we first quantified the amount of diacetyl produced by bacteria using the spectrophotometric method (Mattessich and Cooper 1989). The standard laboratory diet, *E. coli* , did not produce much diacetyl when cultured in LB (Figure 5A). On the other hand, *Lactobacillus paracasei* ( *L. paracasei* ) cultured in MRS medium supplemented with glucose and pyruvate produced diacetyl as previously reported (Figure 5A) (Jyoti et al. 2003). Chemotaxis toward *L. paracasei* depends on *odr‐10* (Choi et al. 2016). To investigate the attraction toward the bacterial odorant, we placed an agar plug with *L. paracasei* on the lid of a chemotaxis assay plate so that animals were not in direct contact with the bacteria (Figure 5B). Day 1 *C. elegans* were attracted toward an agar plug with *L. paracasei* (Figure 5C, wt Day 1) (Choi et al. 2016). Like the diacetyl chemotaxis, chemotaxis toward *L. paracasei* was reduced in the Day 5 wild type, while Day 5 *nhr‐76* mutants maintained the ability to reach *L. paracasei* (Figure 5C). Although *L. paracasei* did not support the growth of *C. elegans* , it supported the growth as a mixture with *E. coli* (Figure 5D). *C. elegans* in a natural habitat is associated with various bacteria, including lactic acid bacteria (Samuel et al. 2016). Thus, they can be attracted to a mixture of bacteria containing diacetyl‐producing bacteria as a food source. Our results suggest that the chemotaxis decline caused by *nhr‐76* possibly reduces the food‐searching behavior of Day 5 animals.

## 3 Discussion

Forward genetic screens for age‐associated phenotypes were limited even in *C. elegans* because animals around Day 5 with a phenotype of interest would have few or no progeny. As a result, their application has only been reported for early‐onset defects like the progressive deficit in locomotion (Kawamura and Maruyama 2019) or increased protein aggregation (Midkiff et al. 2022). Here, we demonstrated that forward genetic screening using Day 4 animals is feasible by mating Day 4 hermaphrodites with Day 1 unmutagenized males. This novel screening strategy enabled us to find an *nhr‐76* mutant, which ameliorates an age‐dependent behavioral defect. *nhr‐76* had a specific age‐dependent role in AWA neurons different from its role in the intestine in Day 1 animals (Noble et al. 2013). Moreover, the *nhr‐76(knj51)* mutation did not reduce fitness, based on the brood size. Thus, *nhr‐76's* function in aging cannot be explained by antagonistic pleiotropy (Austad and Hoffman 2018). Instead, *nhr‐76* appears to actively cause age‐dependent chemotaxis decline. ODR‐10 expression increased from Day 5 to Day 10. The acute downregulation of *odr‐10* might serve to actively decrease chemotaxis ability in Day 5 animals, which retained locomotor ability. In the future, forward genetic screening can be applied to various age‐associated phenotypes manifested at relatively earlier stages (Collins et al. 2008; Stein and Murphy 2012) to reveal active mechanisms of aging.

**FIGURE 6:**
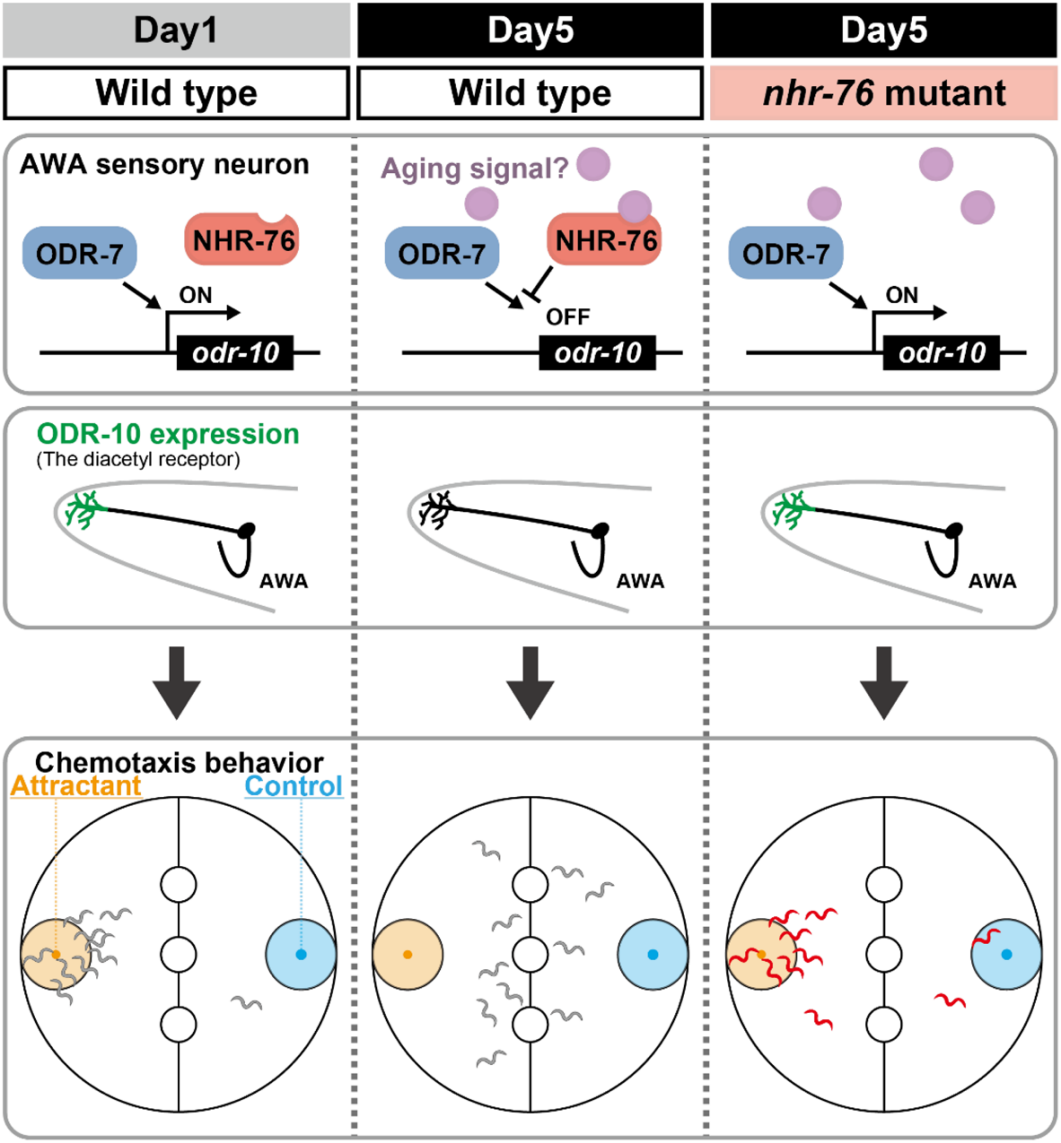
Model of age‐dependent chemotaxis decline. *odr‐10*, encoding diacetyl receptor, is expressed through ODR‐7 in Day 1 animals. In Day 5 animals, NHR‐76 is activated through the ligand‐binding domain by unknown signals shown in purple and inhibits *odr‐10* mRNA expression. The lower ODR‐10 expression results in the decline of chemotaxis behavior toward diacetyl. In *nhr‐76* mutants, the lack of *odr‐10* inhibition maintains the ODR‐10 expression and the chemotaxis behavior in Day 5 animals.

Two nuclear hormone receptors, ODR‐7 and NHR‐76, function as positive and negative regulators of *odr‐10* expression, respectively. Since *odr‐7* affects both diacetyl and pyrazine (Sengupta et al. 1994) and *nhr‐76* only affects diacetyl, NHR‐76 may specifically recognize the *odr‐10* promoter region instead of inhibiting the entire ODR‐7 function (Figure 6). In previous research, a point mutation in the DNA‐binding domain of ODR‐7 caused a defect in chemotaxis toward diacetyl but not toward pyrazine, suggesting that different promoter sequences regulate the expression of the diacetyl receptor and pyrazine receptors (Colosimo et al. 2003). These different promoter sequences may explain why NHR‐76 specifically regulates chemotaxis toward diacetyl. The expression and nuclear localization of NHR‐76 did not change in Day 5 animals. Therefore, we speculate that NHR‐76 is activated during aging (Figure 6). Nuclear hormone receptors can bind to their ligands, such as steroid hormones and hydrophobic vitamins, to change the expression of target genes (Aranda and Pascual 2001). ODR‐7 does not have the LBD (Colosimo et al. 2003), so the activity change is unlikely via ODR‐7. NHR‐76 is homologous to mammalian retinoic acid X receptor (RXR) alpha, whose activity can change with a ligand, such as 9‐cis retinoic acid and docosahexaenoic acid (Evans and Mangelsdorf 2014; Sayers et al. 2022). NHR‐76 might suppress ODR‐7 activity in response to an age‐dependent ligand. In mammals, retinoic acid signaling has been reported to increase with age and to be associated with defective cognitive functions in aged rats (Wołoszynowska‐Fraser et al. 2021). These observations are consistent with our findings and suggest that mammals may share related neuronal aging mechanisms. Although it remains unclear whether *nhr‐76* suppresses *odr‐10* expression in a ligand‐dependent manner, our results linking the nuclear hormone receptor *nhr‐76*, its downstream target *odr‐10*, and a specific behavioral aging phenotype offer a new perspective on active aging (Figure 6). The ligand may function as a systemic “aging signal” to change the activity of multiple nuclear hormone receptors, which link the aging signal to a specific behavior (Figure 6). This hypothesis predicts that other nuclear hormone receptors cause behavioral declines in chemotaxis toward other odorants and other food‐searching behaviors. Indeed, a nuclear hormone receptor, *nhr‐66*, causes the age‐dependent decline in long‐term associative memory in *C. elegans* (Fenyves et al. 2021), and nuclear hormone receptors are proposed to be integrators of sensory cues and internal states (Sural and Hobert 2021).

We showed that *C. elegans* are innately attracted to a diacetyl‐producing lactic acid bacterium, *L. paracasei* . Since the chemotaxis decline is coupled with the end of reproduction (Suryawinata et al. 2024), post‐reproductive individuals might be actively programmed to decrease their attraction to a food source and avoid competing with young animals. Whether aging is genetically “programmed” remains highly controversial. Nevertheless, several programmed‐aging theories propose that senescence frees resources for the next generation, thereby enhancing population fitness (Kowald and Kirkwood 2016)—an idea that resonates with the findings of this study. The active mechanism might also influence associative learning behavior for food because we previously found that aberrant activation of neurons interferes with the intact circuit to cause age‐dependent behavioral decline (Aleogho et al. 2025). Since age‐dependent changes in the nervous system can directly affect the population of a species, an active genetic program might have evolved to couple the end of reproduction with behavioral decline to increase the fitness of a species.

## 4 Materials and Methods

### 4.1 *C. elegans* Culture and Strains

Bristol N2 hermaphrodites were used as the wild‐type *C. elegans* . *C. elegans* were cultured at 23°C and fed with *E. coli* OP50 strain on Nematode Growth Medium (NGM) agar plates (Brenner 1974). OP50 was precultured in LB broth for 16 h at 37°C, and 250 μL was seeded on NGM agar plates. Age‐matched animals were obtained by preparing synchronized eggs with a bleaching solution (1:1 mixture of 1 M NaOH and household bleach) (Stiernnagle 2006). Unless otherwise noted, animals were treated with 25 μM 2′‐Deoxy‐5‐fluorouridine (FUdR) 48 h after preparing eggs to prevent progeny from hatching. The animals grown at 23°C for 3 days, 6 days, and 7 days from eggs were considered Day 1, Day 4, and Day 5 adults, respectively. We used survivors for assays when some animals started dying at Day 10 and Day 15 (Figure 1B–D). The mutants and transgenic strains used in this work are summarized in Table S1. For behavioral assays, transgenic animals carrying extrachromosomal arrays were selected by resistance to 0.125 mg/mL hygromycin on NGM plates from eggs.

### 4.2 Population Chemotaxis Assay

Population chemotaxis assays were conducted at 23°C as previously described (Bargmann et al. 1993; Suryawinata et al. 2024). Synchronized animals were collected with M9 buffer (20 mM KH_2_PO_4_, 20 mM Na_2_HPO_4_, 8 mM NaCl, and 20 mM NH_4_Cl) and washed with M9 buffer once and CTX buffer (5 mM K‐PO_4_, 1 mM CaCl_2_, and 1 mM MgSO_4_) three times. The animals were spotted on the three center circles of a 90‐mm chemotaxis plate containing 12 mL agar (2% Difco agar, 1 mM MgSO_4_, 1 mM CaCl_2_, and 5 mM K‐PO_4_) (Figure 1A). Along with 1 μL of NaN_3_, 4 μL of an attractant diluted with 100% ethanol and 4 μL of 100% ethanol were placed on the centers of the attractant and control circles, respectively (Figure 1A). The assay plates were sealed with parafilm and kept in the dark for 1 h. The animals were killed with chloroform after the assay, and the number of animals in each section was counted under a dissection microscope (Figure 1A, left panel). CTX index was calculated using the formula indicated in Figure 1A. We used approximately 100 animals per assay. In conditions that yielded few progeny (e.g., hygromycin‐resistant transgenic lines), the number of animals occasionally fell below 50; however, each assay included at least 10 animals. Unless otherwise stated, all chemotaxis assays were performed at least nine times over three independent days.

### 4.3 Locomotion Assay

Locomotion speed was measured as the migration distance on a bacterial food lawn at 23°C. A single animal was transferred onto the OP50 lawn of a new NGM plate by picking and removed approximately 1.5 min after transfer. The image of the track on the OP50 lawn was taken with a camera (Panasonic HC‐V620M‐S) and a microscope adapter (MICRONET NY‐VS 811383) under a dissection microscope. The length of a track was measured using ImageJ (Schneider et al. 2012).

### 4.4 Forward Genetic Screen

P_0_ L4 animals were mutagenized with 47 mM EMS for 4 h (Brenner 1974). F_1_ gravid adults were treated with a bleaching solution to obtain synchronized F_2_ eggs. Age‐synchronized F_2_ animals were treated with 25 μM FUdR 48 h after preparing eggs. Twenty‐four hours after FUdR treatment, F_2_ animals were washed off with nematode growth (NG) buffer (51 mM NaCl, 1 mM CaCl_2_, 1 mM MgSO_4_, and 25 mM K‐PO_4_) and transferred onto new NGM plates with OP50 without FUdR to remove mutants with developmental defects. The chemotaxis assay toward 0.01% diacetyl was conducted with Day 4 F_2_ animals (6 days after preparing eggs) for 30 min without NaN_3_. The attracted Day 4 F_2_ animals were pooled, transferred to new NGM plates, and mated with Day 1 wild‐type males. These animals were considered P_0_ of the next round of the screening, and the process of chemotaxis and mating was repeated to reduce false positives and enrich mutants until the CTX index became significantly high (Figure 1A). After this cycle, 8 to 16 F_2_ animals were singly isolated, and the chemotaxis ability was tested on Day 4 and Day 5. A mutant consistently showing a high CTX index was named *knj39*.

### 4.5 Quantitative RT‐PCR

Synchronized animals were used for RNA extraction and cDNA synthesis. Animals grown at 23°C for 56 h (non‐gravid adults), instead of 72 h for the chemotaxis assay, were considered Day 1 to exclude the contribution of eggs. For each biological replicate, we pooled at least 1000 synchronized animals for RNA extraction. RNAiso plus (Takara Bio) and ReverTra Ace (TOYOBO) were used for RNA extraction and cDNA synthesis, respectively. Quantitative polymerase chain reaction (qPCR) was performed with THUNDERBIRD SYBR qPCR Mix (TOYOBO) using LightCycler 96 (Roche) and gene‐specific primers described in Table S2. *cdc42*, showing stable expression during aging, was used as a reference (Mann et al. 2016). The mean value of three technical replicates was considered the value for one biological replicate, and three or more biological replicates were examined for one condition.

### 4.6 Confocal Microscopy

Animals were immobilized in a drop of M9 containing 5 mM levamisole on a 4% agarose pad. Images were taken with a confocal microscope LSM880 (Zeiss) with 488‐nm and 561‐nm lasers for GFP and tagRFP, respectively. The positions of AWA sensory neurons were determined by *odr‐10*p::tagRFP. Fluorescence intensities of the nucleus and cytosol were measured for NHR76::GFP. On the other hand, we measured ODR‐10::GFP intensity in the amphid region to minimize artifactual signals from mislocalized or aggregated proteins. Images were quantified using ImageJ (Schneider et al. 2012).

### 4.7 Lifespan Assay

Animals were treated with or without 25 μM FUdR starting from 48 h after preparing eggs. Twenty‐five animals were transferred to individual plates on Day 1. The animals that did not respond to touch were considered dead and counted every other day; the animals that escaped from the plates were considered censored. After scoring, all living animals were transferred to a new NGM plate with or without 25 μM FUdR by picking. The analyses were conducted with OASIS2 (online application for survival analysis 2) (Han et al. 2016).

### 4.8 Plasmids

Gateway system (Thermo Fisher Scientific) was used to generate expression plasmids from the entry clone and destination plasmids using the LR reaction. Entry clones and destination plasmids were generated by Gibson assembly (Gibson et al. 2009). Plasmids were summarized in Table S3.

### 4.9 CRISPR Genome Editing and Transgenic Strains

CRISPR KO strains of *nhr‐76* were generated by following the co‐CRISPR strategy (Kim et al. 2014) using two *nhr‐76* crRNAs, *dpy‐10* crRNA, tracrRNA, and Cas9 protein. *nhr‐76* deletions were detected by PCR and confirmed by Sanger sequencing. Deletion alleles of LBD were also generated by the same strategy as CRISPR KO. The mRNA expression level and sequence were confirmed by qPCR and Sanger sequencing, respectively. Transgenic animals were generated by microinjection following the standard method (Mello et al. 1991). For the transgenic rescue experiments, PCR products of an *nhr‐76* genomic fragment at 5 ng/μL (*nhr‐76p*) or plasmids of the *nhr‐76* cDNA under the tissue/cell‐specific promoters (*ges‐1*p, *rgef‐1*p, or *odr‐10*p) at 25 ng/μL were injected into NUJ571 *nhr‐76(knj51)*. For the overexpression experiment, *nhr‐76* cDNA under the AWAspecific *odr‐10* or *odr‐7* promoter at 50 ng/μL was injected into N2. For the behavioral assays, co‐injection markers (*rps‐0*p::HygR at 10 ng/μL and coelomocyte RFP at 25 ng/μL) were injected together with each plasmid. Transgenic animals carrying extrachromosomal arrays were selected by resistance to 0.125 mg/mL hygromycin on the NGM plate and confirmed by the red fluorescence in coelomocytes. At least two transgenic lines were tested. For confocal imaging, tagRFP under the AWA‐specific *odr‐10* promoter at 25 ng/μL was injected into N2 with a co‐injection marker (pRF4 *rol6(su1006)* at 25 ng/μL). For all injections, an empty pUC19 plasmid was used to adjust the total concentration of the injected DNA to 100 ng/μL. The primers and crRNAs used in these experiments are described in Table S2.

### 4.10 Diacetyl Quantification

Diacetyl quantification was performed as previously described (Mattessich and Cooper 1989). *L. paracasei* was precultured in MRS (BD Difco *Lactobacilli* MRS Broth), minimal MRS (5.0 g Yeast extract, 0.1 g MgSO_4_·7H_2_O, 0.07 g MnSO_4_·5H_2_O, 2.0 g NaH_2_PO_4_, 1.0 g Tween‐80 in 1 L of MilliQ water, pH was adjusted to 5.5), Glucose and citrate‐supplemented (6.3 g Glucose and 2.5 g Trisodium citrate dihydrate) minimal MRS, or Glucose and pyruvate‐supplemented (6.7 g Glucose and 2.12 g Sodium pyruvate) minimal MRS (Jyoti et al. 2003). Precultured *L. paracasei* were seeded on 1.5% agar in the respective media and incubated for 3 days at 37°C. Two solutions, 325 μL of saturated creatine (0.2 g of creatine into 10 mL of Milli‐Q water) and 150 μL of Solution B (3% (w/v) NaOH, 3.5% (w/v) α‐naphthol), were mixed in a 1.5 mL tube right before the assay. Agar plugs of the same size, with and without bacteria, were cut out with a P1000 tip, added to the solution above, and crushed well with a P1000 tip. After mixing with a vortex mixer for 15 s, the solution was centrifuged at 10,000 rpm for 1 min at room temperature and filtered through a 0.22 μm MILLEX GV filter (0.22 μm, Millipore) to remove the agar. The solution was left at room temperature for 30 min, and 525‐nm absorbance was measured using the DS‐11 spectrophotometer (DeNovix).

### 4.11 Population Chemotaxis Assay for Bacteria

The population chemotaxis assay toward bacterial odorants was based on the population chemotaxis assay described above, except that bacterial agar plugs instead of chemicals were used as an attractant. *L. paracasei* was prepared using the same method as diacetyl quantification, using agar plates with glucose and pyruvate‐supplemented minimal MRS. Cultured plates with bacteria were equilibrated at room temperature in a box before the chemotaxis assay. Agar plugs with and without bacteria were cut out with a P1000 tip and placed on the lid of the attractant and control sides of an assay plate, respectively.

### 4.12 Brood Size Measurement

P_0_ L4 animals were transferred to individual plates 48 h after egg preparation and transferred to a new plate daily until they stopped depositing eggs. The number of F_1_ progeny was counted under a dissection microscope when they became adults. To calculate the brood size ratio of an animal, the number of progeny produced on each day was divided by the total number of progeny of the animal.

### 4.13 *C. elegans* Growth on *L. paracasei*

*E. coli* OP50 and *L. paracasei* were cultured for 16 h at 37°C with LB broth and MRS, respectively. The cultured bacteria were centrifuged at 7000 rpm for 10 min at 4°C. The pellets were resuspended with 0.9% NaCl and centrifuged at 7000 rpm for 10 min at 4°C. Resuspension and centrifugation were repeated once more, and the supernatants were removed.

The pellets were diluted with Nematode Growth buffer (NG buffer, 3 g NaCl, 1 mL 1 M CaCl_2_, 1 mL 1 M MgSO_4_, and 25 mL 1 M K‐PO_4_ in 1 L of Milli‐Q water) to 100 mg/mL. Two hundred microliters of 100 mg/mL bacteria were seeded on peptone‐free NGM plates. In the mixed bacterial food condition, *E. coli* and *L. paracasei* were mixed at the same ratio immediately before seeding. The seeded plates were dried for 24 h. Synchronized eggs were placed on the bacterial plates, and the growth of animals was observed 72 h later at 23°C. The images were taken using the same method as the locomotion assay.

### 4.14 Statistical Analysis

Graphs were generated using R (R_Core_Team 2020). Statistical analyses were performed in R or EZR (Kanda 2013). For assays with two factors, we used two‐way ANOVA after checking assumptions (Shapiro–Wilk for normality; Brown–Forsythe for homogeneity of variances). When assumptions were violated, we conducted sensitivity analyses using generalized least squares (GLS) and HC3‐robust F. We confirmed that conclusions were unchanged across two‐way ANOVA, GLS, and HC3‐robust F. Other behavioral data were analyzed with nonparametric tests (Mann–Whitney *U* for single comparisons; Kruskal–Wallis for multiple groups). Parametric tests were used for datasets from non‐behavioral assays or for those with only three samples (Student's *t*‐test for single comparisons; one‐way ANOVA for multiple groups). In box and whisker plots, the box, vertical line, and whiskers indicate the first and third quartiles, the median, and the maximum and minimum values excluding outliers, respectively. In bar plots, the standard error of the mean is indicated as a black line.

## Author Contributions

K.N. was involved in conceptualization, funding acquisition, project administration, and supervision. R.Y. and K.N. were involved in methodology, investigation, writing – original draft, and writing – review and editing. R.Y. was involved in visualization.

## Conflicts of Interest

The authors declare no conflicts of interest.

## Supporting information


**Figure S1:** GFP‐tagged ODR‐10 fluorescence did not decline from Day 1 to Day 5 in the *knj39* mutants.
**Figure S2:** Aged animals show locomotion defects.
**Figure S3:** Transient FUdR treatment and mating increase the number of progeny in aged animals.
**Figure S4:** Survival curve of the wild type and *knj39* mutants in the no‐FUdR condition.
**Figure S5:** *nhr‐76(knj51)* mutants have normal reproduction.
**Figure S6:** Aged *nhr‐76* mutants require *odr‐10* for diacetyl chemotaxis.
**Figure S7:** *nhr‐76* mRNA expression in 7 alleles of *nhr‐76* mutants.
**Figure S8:** *nhr‐76* mutations are semidominant.
**Figure S9:** The quantification of chemotaxis ability toward 0.1% diacetyl.
**Figure S10:** Tissue/cell‐specific rescue experiment for *nhr‐76(knj51)* mutants.
**Figure S11:** Mutants involved in lipid metabolism do not ameliorate age‐dependent chemotaxis decline.
**Figure S12:** *nhr‐76* overexpression does not decrease the chemotaxis ability on Day 1.
**Figure S13:** Transcripts of LBD‐deleted *nhr‐76* mRNAs.
**Table S1:** *C. elegans* strains.
**Table S2:** Oligonucleotides. Primers and crRNAs.
**Table S3:** Plasmids.

## Data Availability

The data that supports the findings of this study is available in the Appendix S1 of this article.
