# Supplementary material for: A Nuclear Hormone Receptor nhr‐76 Induces Age‐Dependent Chemotaxis Decline in C. elegans
Source: Aging Cell. 2025 Oct 23;24(12):e70277. doi: 10.1111/acel.70277 (PMC12686552; doi:10.1111/acel.70277)
Supplement: Supplementary file 1 — Figure S1: GFP‐tagged ODR‐10 fluorescence did not decline from Day 1 to Day 5 in the knj39 mutants. Figure S2: Aged animals show locomotion defects. Figure S3: Transient FUdR treatment and mating increase the number of progeny in aged animals. Figure S4: Survival curve of the wild type and knj39 mutants in the no‐FUdR condition. Figure S5: nhr‐76(knj51) mutants have normal reproduction. Figure S6: Aged nhr‐76 mutants require odr‐10 for diacetyl chemotaxis. Figure S7: nhr‐76 mRNA expression in 7 alleles of nhr‐76 mutants. Figure S8: nhr‐76 mutations are semidominant. Figure S9: The quantification of chemotaxis ability toward 0.1% diacetyl. Figure S10: Tissue/cell‐specific rescue experiment for nhr‐76(knj51) mutants. Figure S11: Mutants involved in lipid metabolism do not ameliorate age‐dependent chemotaxis decline. Figure S12: nhr‐76 overexpression does not decrease the chemotaxis ability on Day 1. Figure S13: Transcripts of LBD‐deleted nhr‐76 mRNAs. Table S1: C. elegans strains. Table S2: Oligonucleotides. Primers and crRNAs. Table S3: Plasmids. [file ACEL-24-e70277-s001.zip › acel70277-sup-0001-FigureS1-S13-TableS1-S3@NLM4_Manuscript_v55_AgingCellsup_Clean.pdf]

A nuclear hormone receptor *nhr-76* induces age-dependent chemotaxis decline in *C. elegans*

Rikuou Yokosawa[1] and Kentaro Noma[1]*

[1]Graduate School of Science, Nagoya University; Nagoya, 464-8602, Japan.

*Kentaro Noma
Email: noma.kentaro.f1@f.mail.nagoya-u.ac.jp

This PDF file includes:

Figures S1 to S13
Tables S1 to S3

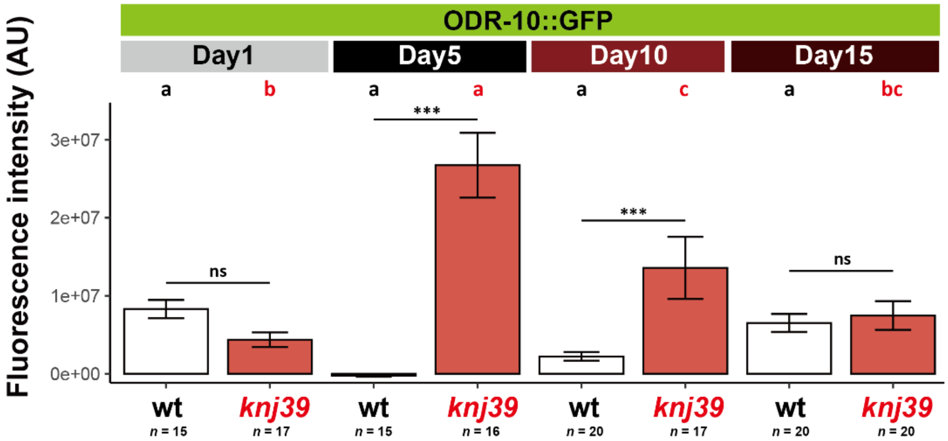

Figure S1 The quantification of the fluorescence intensity of ODR-10::GFP reporter (*kyIs53[odr-10::GFP]*) in the amphid region, shown in Figure 1D. Statistical tests were conducted using two-way ANOVA with Tukey's test. ns: p >0.05; ***p <0.001. Different letters indicate significant pairwise differences within the same genotype (p <0.05).

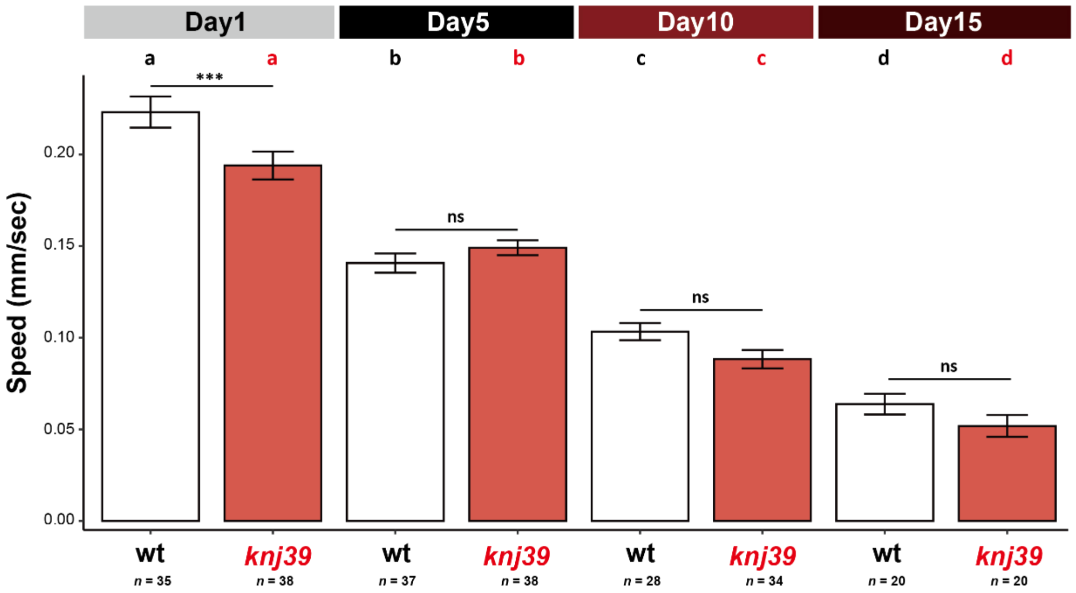

Figure S2 Aged animals show locomotion defects. The locomotion speed of Day1 to Day15 animals was measured on food for the wild type and *knj39* mutants. Statistical tests were conducted using two-way ANOVA with Tukey's test. ns: $p > 0.05$; ***$p < 0.001$. Different letters indicate significant pairwise differences within the same genotype ($p < 0.05$).

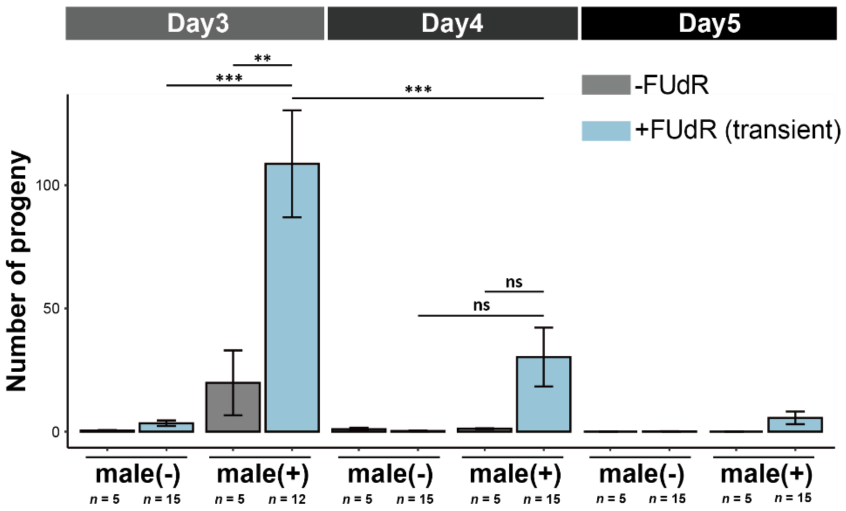

Figure S3 Transient FUdR treatment and mating increase the number of progeny in aged animals. The total number of progeny produced by Day 3, 4, and 5 animals. For the FUdR condition, animals were treated with FUdR for 24 hours from L4 (48 hours after epp preparation). For the condition with males, an excess number of young males were added to Day3, Day4, or Day5 hermaphrodites. Statistical tests were conducted using One-way ANOVA with Tukey's test. ns: $p > 0.05$; ** $p < 0.01$; *** $p < 0.001$.

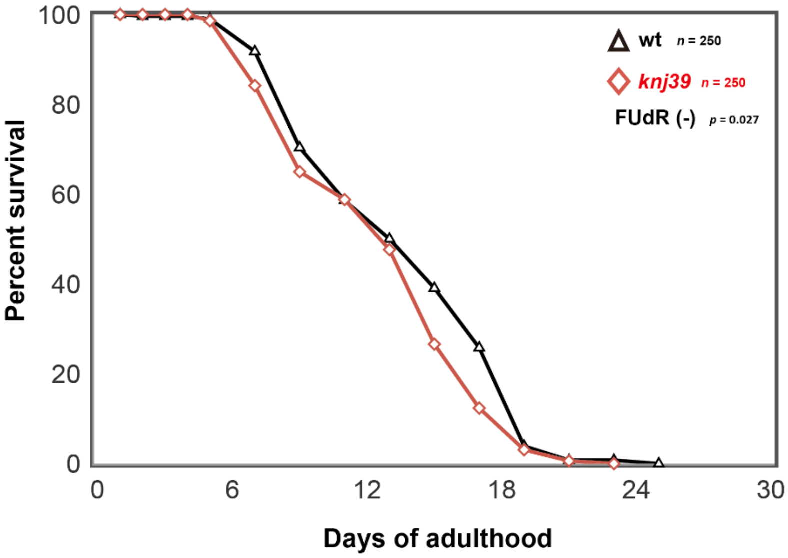

Figure S4 Survival curve of the wild type and *knj39* mutants in the no-FUdR condition. Statistical tests were conducted using Log-rank test.

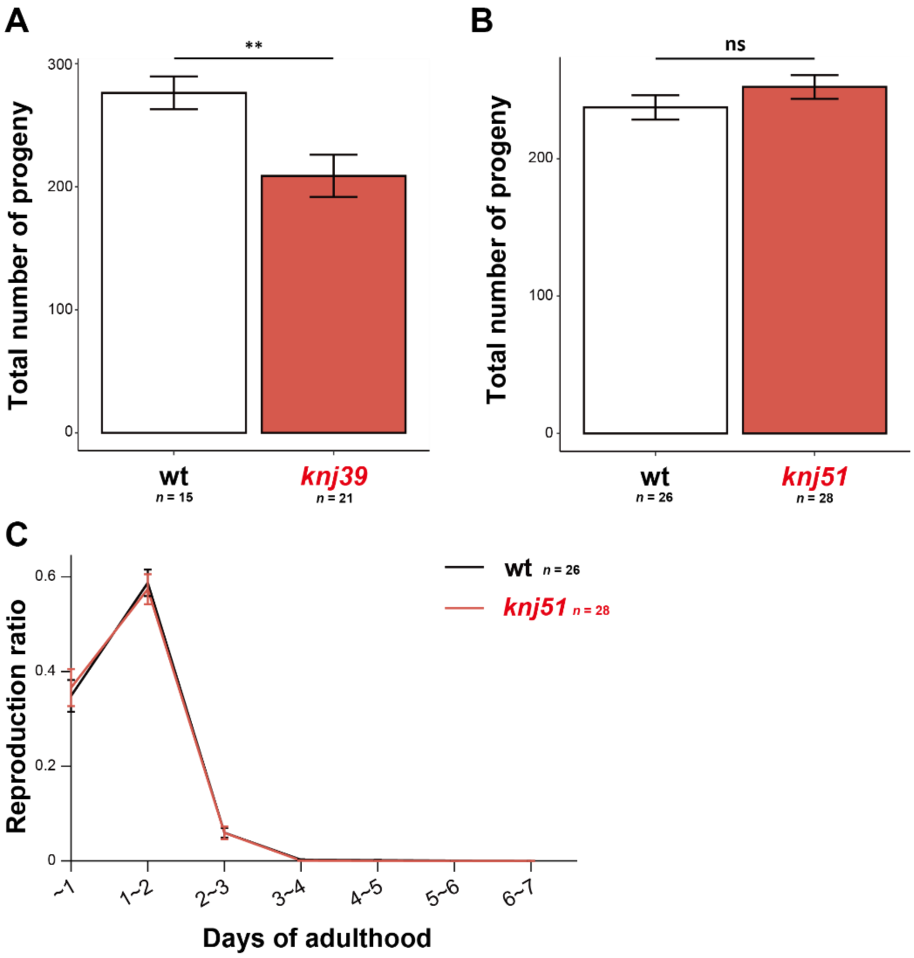

Figure S5 *nhr-76(knj51)* mutants have normal reproduction. (A) and (B) The total number of progeny of the wild type and *nhr-76* mutants (*knj39* in (A) and *knj51* in (B)). (C) The reproductive span of the wild-type and *nhr-76(knj51)* mutants. The number of progeny deposited during the indicated period was normalized with the total number of progeny of each animal. Statistical tests were conducted using Student's t-test for (A) and (B). ns: $p > 0.05$; ** $p < 0.01$.

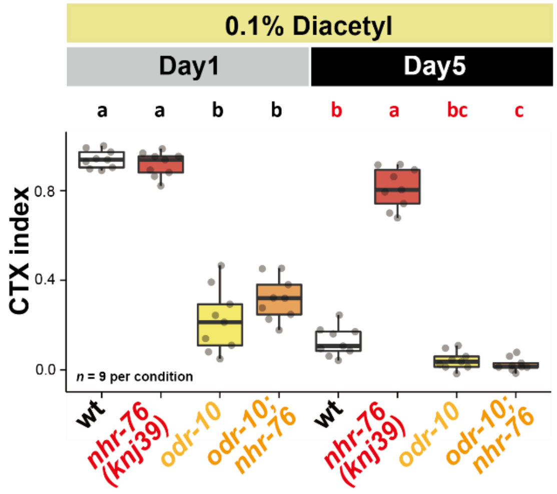

Figure S6 Aged *nhr-76* mutants require *odr-10* for diacetyl chemotaxis. The quantification of chemotaxis ability toward 0.1% diacetyl for the wild type, *nhr-76(knj39)*, *odr-10(ky225)*, and *nhr-76(knj39); odr-10(ky225)*. Day1 and Day5 animals were tested for each genotype. Statistical tests were conducted using two-way ANOVA with Tukey's test. Different letters indicate significant pairwise differences within the same genotype (p <0.05).

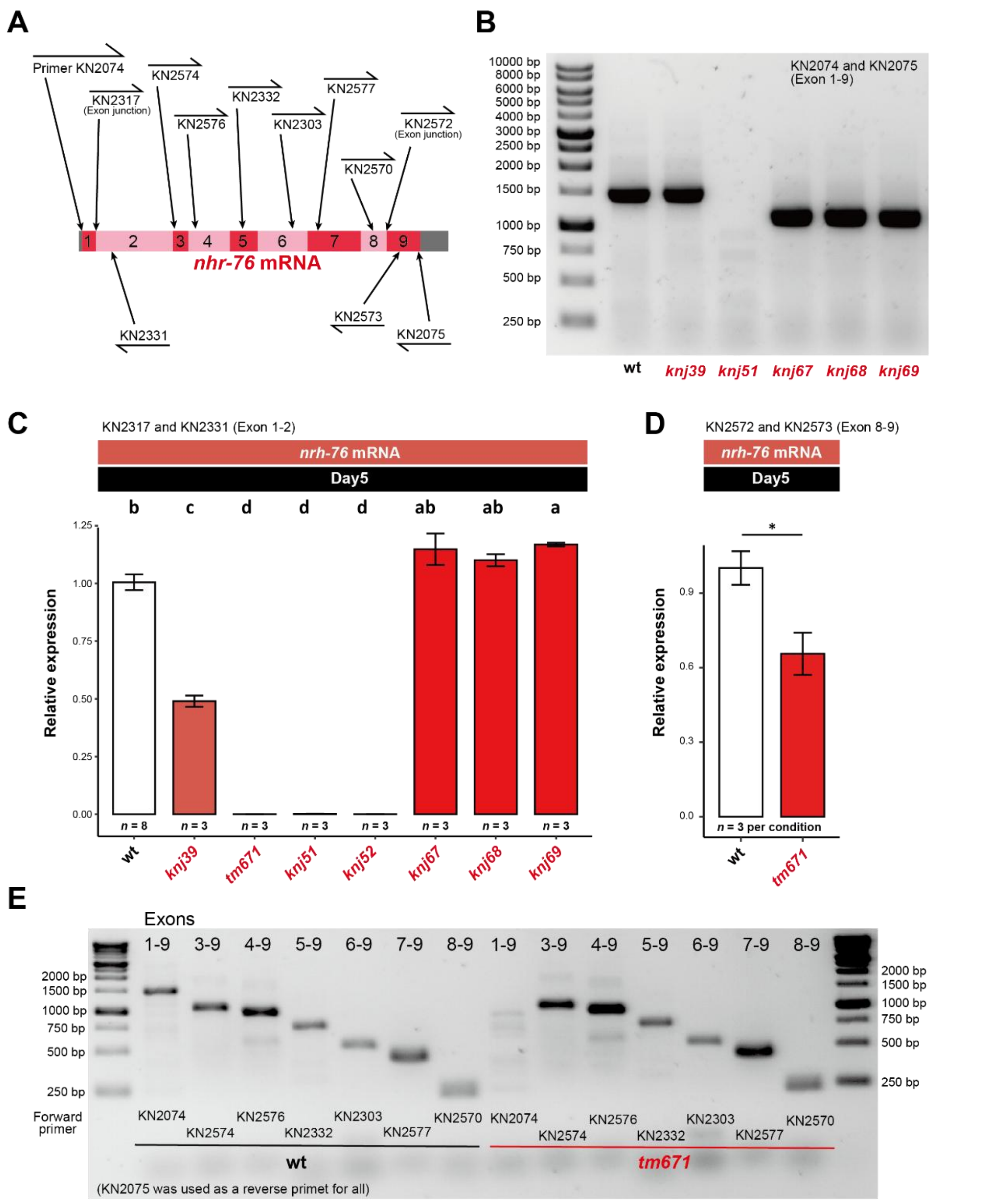

Figure S7 (A) Schematic of *nhr-76* mRNA with the primers used in (B)-(E). Exon numbers are shown in the boxes. (B) Image of agarose gel electrophoresis for the RT-PCR products using *nhr-76* cDNA and primers targeting the first and last exons of the *nhr-76* transcript (KN2074 and KN2075). (C) and (D) The mRNA expression of *nhr-76,* normalized by the mean value of the Day5 wild type. Error bars indicate SEM. (E) Image of agarose gel electrophoresis for the RT-PCR products. The forward primers targeting exon 1, 3, 4, 5, 6, 7, or 8

were used with the reverse primer targeting exon 9 of the *nhr-76* transcript. Statistical tests were conducted using one-way ANOVA with Tukey's test for (C); Student's t-test for (D). **p* <0.05. Different letters indicate significant pairwise differences ($p < 0.05$).

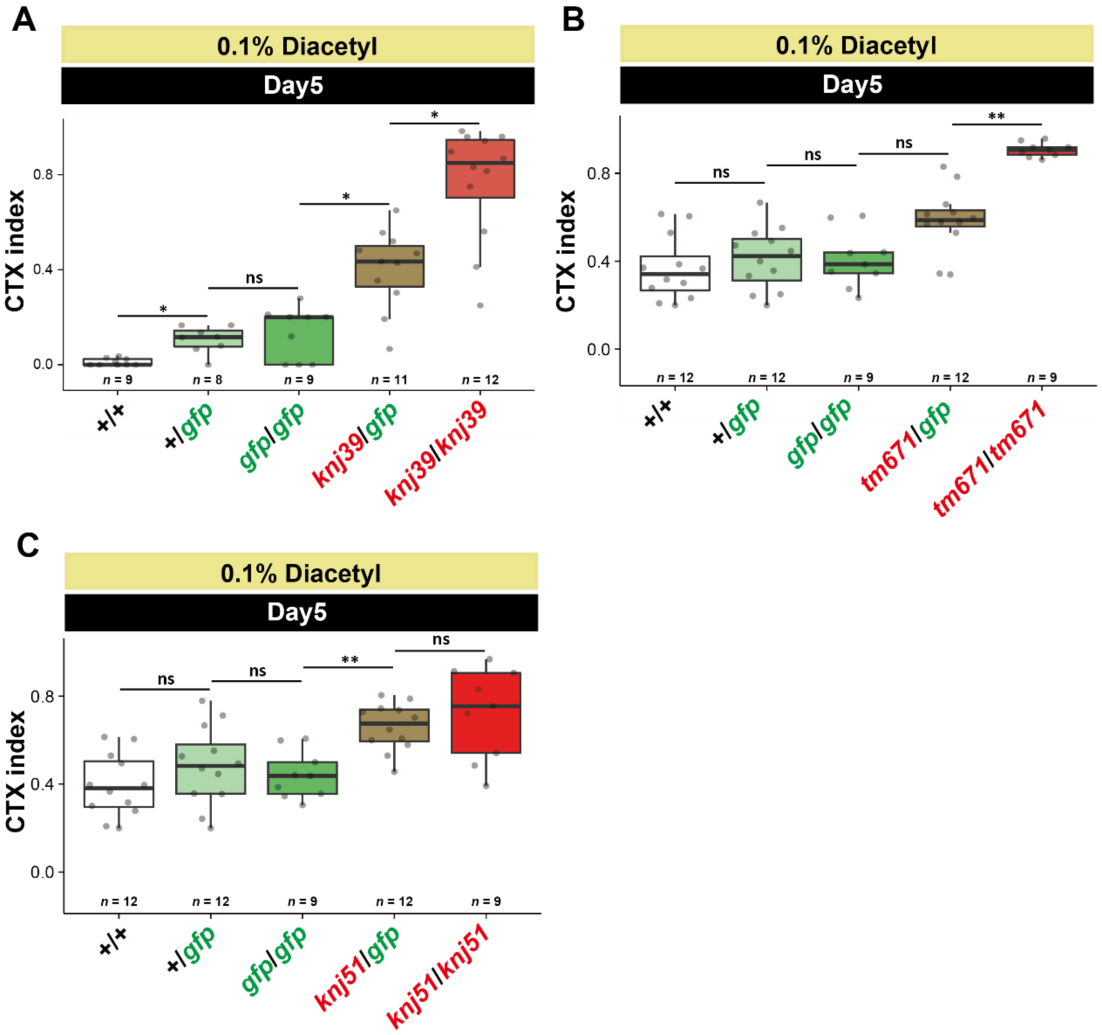

Figure S8 *nhr-76* mutations are semidominant. The quantification of chemotaxis ability toward 0.1% diacetyl for the wild type, a GFP reporter strain (CZ10969 *muIs32[mec-7p::gfp]*), *nhr-76* mutants, and the crossed F_1 animals. After crossing wild-type (+/+) or *nhr-76* mutants with CZ10969 males carrying the GFP marker (*gfp*), GFP-positive F_1 animals were counted as the heterozygotes in chemotaxis assays. (A) *nhr-76(knj39)*. (B) *nhr-76(tm671)*. (C) *nhr-76(knj51)*. Statistical tests were conducted using the Kruskal-Wallis with Steel-Dwass test. ns: $p > 0.05$; *$p < 0.05$; **$p < 0.01$.

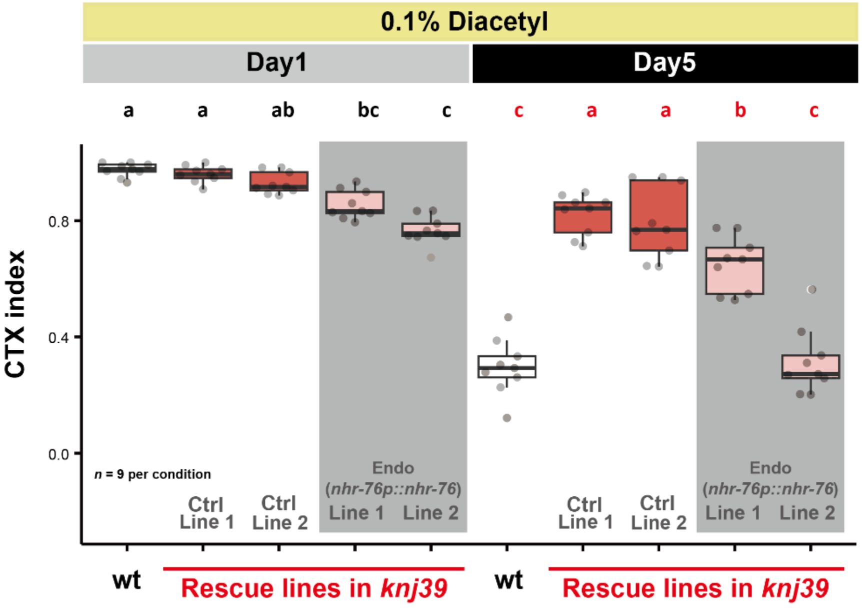

Figure S9 The quantification of chemotaxis ability toward 0.1 % diacetyl. The wild type, *nhr-76* (*knj39*) mutants, and the transgenic lines expressing the PCR fragment of *nhr-76* locus were tested. The control strains (Ctrl) carry only the co-injection markers (HygR and coelomocyte - RFP). All the transgenic strains were treated with hygromycin from eggs. Statistical tests were conducted using two-way ANOVA with Tukey's test. Different letters indicate significant pairwise differences within the same age group (p <0.05).

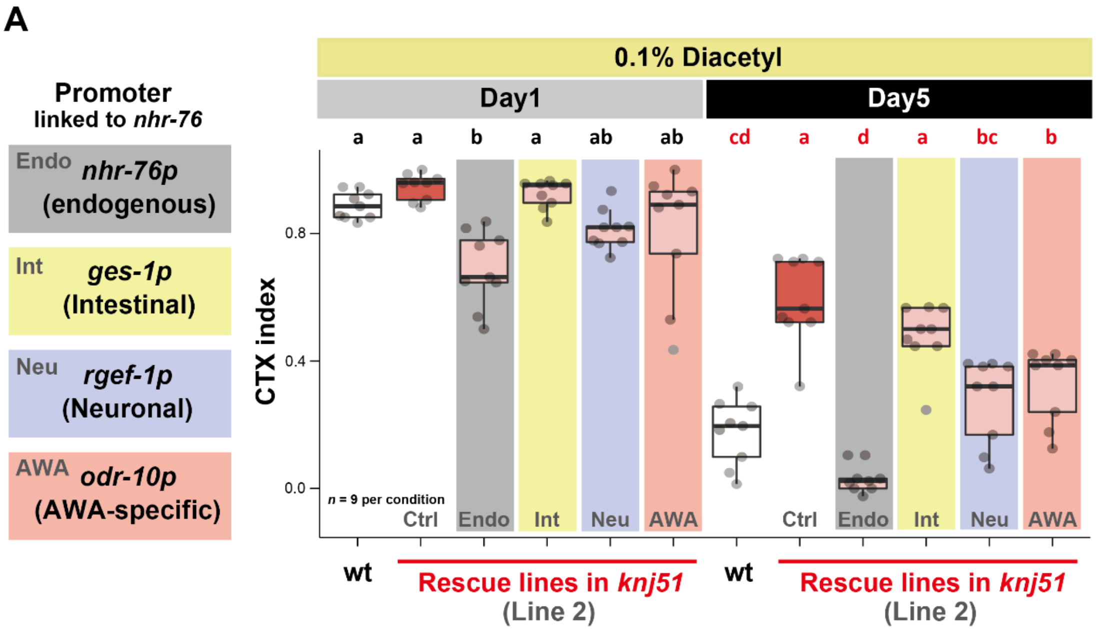

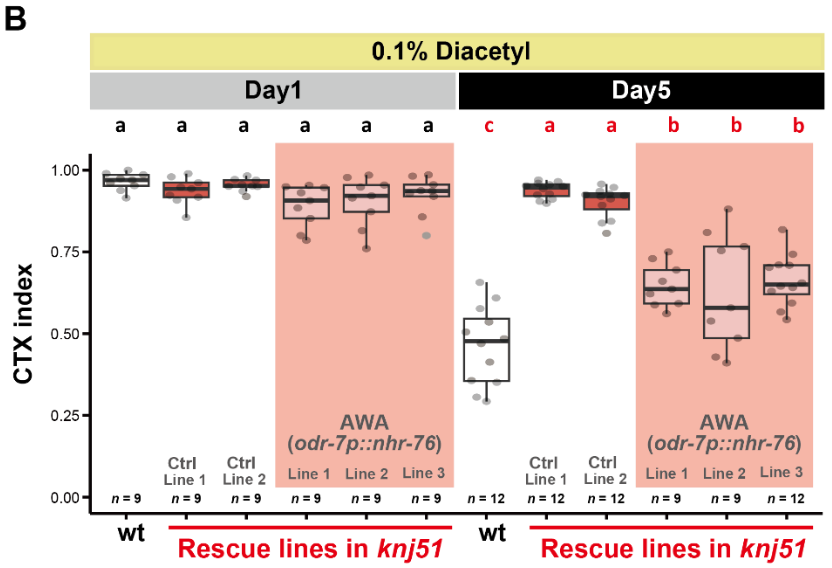

Figure S10 Tissue/cell-specific rescue experiment for *nhr-76(knj51)* mutants. The quantification of chemotaxis ability toward 0.1 % diacetyl. The control strains (Ctrl) carry only the co-injection markers (HygR and coelomocyte -RFP). All the transgenic strains were treated with hygromycin from eggs. (A) The *nhr-76* rescue lines, which are different from Figure 2C, were tested (Line 2). (B) AWA-specific rescue with the *nhr-76* expression under the control of *odr-7* promoter (*odr-7*p). Statistical tests were conducted using two-way ANOVA with Tukey's test. Different letters indicate significant pairwise differences within the same age group (p <0.05).

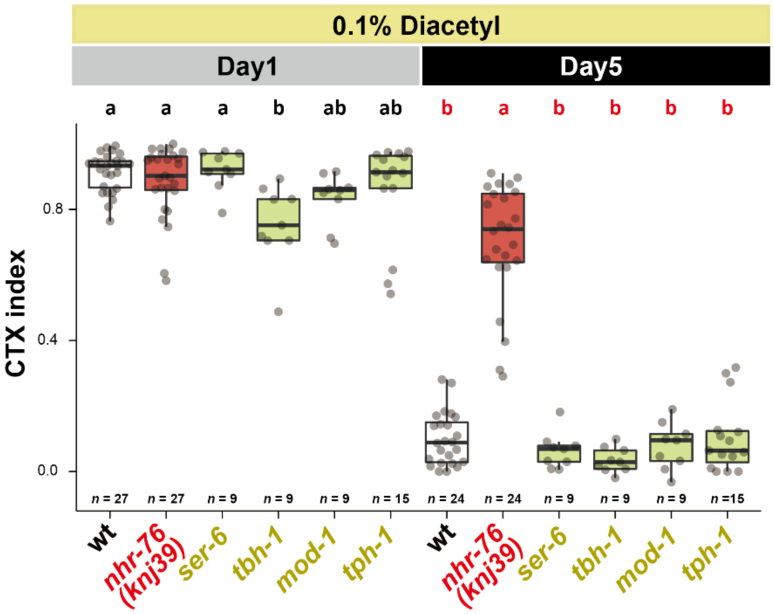

Figure S11 Mutants involved in lipid metabolisms do not ameliorate age-dependent chemotaxis decline. The quantification of chemotaxis ability toward 0.1% diacetyl. Chemotaxis assays were conducted with indicated genotypes and ages. Statistical tests were conducted using two-way ANOVA with Tukey's test. Different letters indicate significant pairwise differences within the same age group (p <0.05).

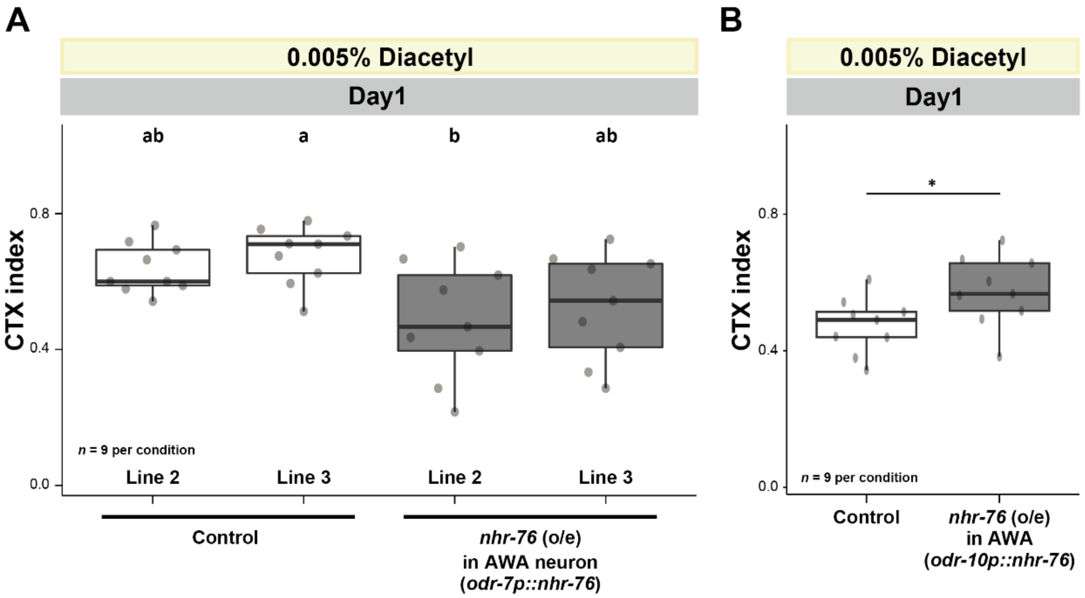

Figure S12 *nhr-76* overexpression does not decrease the chemotaxis ability on Day1. The quantification of chemotaxis ability toward 0.005% diacetyl, which is around EC50 for wild-type Day1 animals. (A) The same chemotaxis assays in Fig. 4B were conducted with different transgenic lines in the wild-type background (Lines 2 and 3). (B) The *nhr-76* overexpression line with the *odr-10* promoter. The same transgene as Line 1 of the AWA-specific rescue in Figure 2C was used in the wild-type background. Statistical tests were conducted using the Kruskal-Wallis with Steel-Dwass test for (A); Mann-Whitney U test for (B). *$p < 0.05$. Different letters indicate significant pairwise differences ($p < 0.05$).

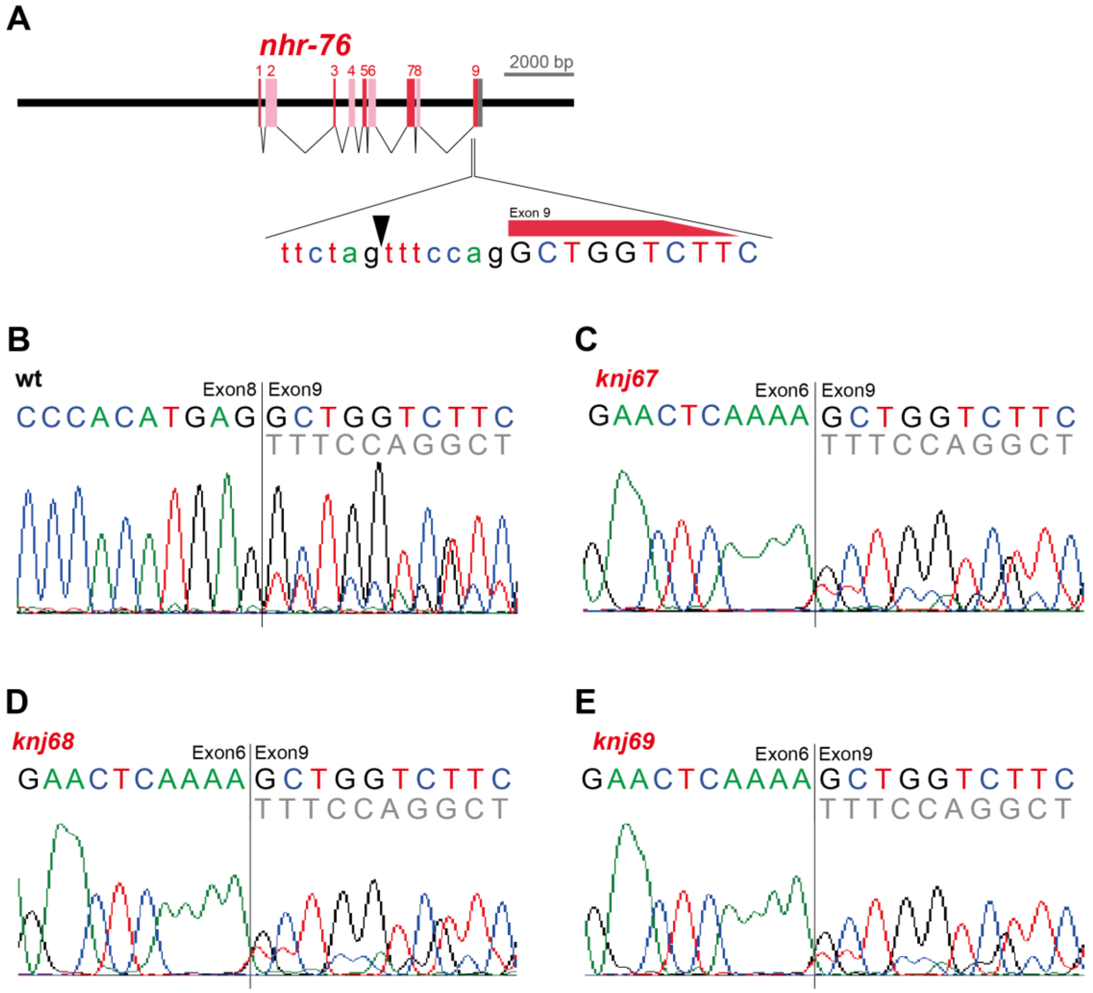

Figure S13 Transcripts of LBD-deleted *nhr-76* mRNAs. (A) Schematic of a gene structure and sequence of *nhr-76*. An alternative splicing site is indicated in the black arrowhead. (B)-(E) The peaks of Sanger sequencing around the exon-exon junctions of the wild type and LBD-deleted mutants (*knj67*, *knj68*, and *knj69*). We note that the peaks are doubled after the exon8-exon9 junction, indicating a possible alternative splicing that adds 7 nucleotides before exon 9. This possible alternative splicing was detected similarly in the wild type and LBD-deleted mutants.

Table S1 *C. elegans* strains.

Table S1 *C. elegans* strains

Strain	Genotype	Note	Source
N2	*wild type*		CGC
NUJ392	*nhr-76(knj39) IV*	2x outcrossed *knj39*	This study, from forward genetic screen
NUJ455	*nhr-76(knj39) IV*	3x outcrossed *knj39*	This study, Cross
NUJ553	*nhr-76(knj39) IV*	4x outcrossed *knj39*	This study, Cross
NUJ557	*nhr-76(knj39) IV*	5x outcrossed *knj39*	This study, Cross
NUJ580	*kyIs53[odr-10::GFP] X*	4x outcrossed strain of CX3344 *kyIs53[odr-10::GFP] X*	This study, Cross
NUJ648	*nhr-76(knj39) IV; kyIs53[odr-10::GFP] X*		This study, Cross
NUJ560	*nhr-76(tm671) IV*	2x outcrossed strain of *nhr-76(tm671)* from NBRP	This study, Cross
NUJ571	*nhr-76(knj51) IV*		This study, CRISPR
NUJ603	*nhr-76(knj52) IV*		This study, CRISPR
NUJ588	*nhr-76(knj51) IV; knjEx235[ccRFP + rps-0p::HygR]*	Control for the co-injection markers (Hygromicine resistantce and Coelomocyte RFP) (Line1)	This study, Injection
NUJ576	*nhr-76(knj51) IV; knjEx226[nhr-76(gDNA) + ccRFP + rps-0p::HygR]*	Rescue of *nhr-76* with a PCR-amplified genomic fragment (Line1)	This study, Injection and Cross
NUJ607	*nhr-76(knj51) IV; knjEx248[ges-1p::nhr-76(cDNA) + ccRFP + rps-0p::HygR]*	Intestinal rescue of *nhr-76* (Line1)	This study, Injection
NUJ605	*nhr-76(knj51) IV; knjEx246[rgef-1p::nhr-76(cDNA) + ccRFP + rps-0p::HygR]*	Pan-neuronal rescue of *nhr-76* (Line1)	This study, Injection
NUJ609	*nhr-76(knj51) IV; knjEx250[odr-10p::nhr-76(cDNA) + ccRFP + rps-0p::HygR]*	AWA-specific rescue of *nhr-76* (Line1)	This study, Injection
CX4	*odr-7(ky4) X*		CGC
NUJ624	*nhr-76(knj39) IV; odr-7(ky4) X*		This study, Cross
NUJ636	*wgIs203[nhr-76::TY1::EGFP::3xFLAG + unc-119(+)]; knjEx93[odr-10p::tagRFP + rol-6(su1006)]*	*knjEx93* was crossed with OP203 *unc-119 (tm4063) III; wgIs203 [nhr-76::TY1::EGFP::3xFLAG + unc-119(+)]* from CGC. It may contain *unc-119(tm4063)* .	This study, Cross
NUJ627	*knjEx234[ccRFP + rps-0p::HygR]*	Co-injection marker control (Hygromicine resistantce and Coelomocyte RFP) (Line1)	This study, Cross
NUJ634	*knjEx261[odr-7p::nhr-76(cDNA) + ccRFP + rps-0p::HygR]*	AWA-specific *nhr-76* over expression (Line1)	This study, Injection
CZ10969	*muIs32[mec-7p::GFP + lin-15(+)] II*	Used for ganerating heterozygous animals in dominancy test	Ch'ng, Q et al. (2003) Geneteics
NUJ457	*odr-10(ky225) X*	1x outcrossed strain of CX3410 *odr-10(ky225)X* from CGC	This study, Cross
NUJ623	*nhr-76(knj39) IV; odr-10(ky225) X*		This stury, Cross
NUJ587	*nhr-76(knj51) IV; knjEx234[ccRFP + rps-0p::HygR]*	Co-injection marker control (Hygromicine resistantce and Coelomocyte RFP) (Line 2)	This study, Injection
NUJ590	*nhr-76(knj51) IV; knjEx227[hr-76(gDNA) + ccRFP + rps-0p::HygR]*	Genomic fragment rescue of *nhr-76* (Line2)	This study, Injection and Cross
NUJ608	*nhr-76(knj51) IV; knjEx249[ges-1p::nhr-76(cDNA) + ccRFP + rps-0p::HygR]*	Intestin-specific rescue of *nhr-76* (Line2)	This study, Injection
NUJ612	*nhr-76(knj51) IV; knjEx253[rgef-1p::nhr-76(cDNA) + ccRFP + rps-0p::HygR]*	Pan-neuronal rescue of *nhr-76* (Line2)	This study, Injection
NUJ611	*nhr-76(knj51) IV; knjEx252[odr-10p::nhr-76(cDNA) + ccRFP + rps-0p::HygR]*	AWA-specific rescue of *nhr-76* (Line2)	This study, Injection
IK1348	*ser-6(tm2146) IV*	2x outcrossed *ser-6(tm2146)* from NBRP	A gift from Ikue Mori
MT9455	*tbh-1(n3247) X*		CGC
MT9668	*mod-1(ok103) V*		CGC
NUJ578	*tph-1(mg280) II*	1x backcrossed strain of MT15434 *tph-1(mg280) II* from CGC	This study, Cross
NUJ628	*knjEx235[ccRFP + rps-0p::HygR]*	Co-injection marker control (Hygromicine resistantce and Coelomocyte RFP) (Line 2)	This study, Injection and Cross
NUJ629	*knjEx236[ccRFP + rps-0p::HygR]*	Co-injection marker control (Hygromicine resistantce and Coelomocyte RFP) (Line 3)	This study, Injection and Cross
NUJ633	*knjEx260[odr-7p::nhr-76(cDNA) + ccRFP + rps-0p::HygR]*	AWA-specific *nhr-76* over expression (Line2)	This study, Injection
NUJ635	*knjEx262[odr-7p::nhr-76(cDNA) + ccRFP + rps-0p::HygR]*	AWA-specific *nhr-76* over expression (Line3)	This study, Injection
NUJ721	*nhr-76(knj67) IV*		This study, CRISPR
NUJ722	*nhr-76(knj68) IV*		This study, CRISPR
NUJ723	*nhr-76(knj69) IV*		This study, CRISPR
NUJ791	*nhr-76(knj51)IV; knjEx261[odr-7p::nhr-76(cDNA) + ccRFP + rps-0p::HygR]*	AWA-specific *nhr-76* over expression with the *odr-7* promoter in the *nhr-76(knj51)* background (Line1)	This study, Injection
NUJ790	*nhr-76(knj51)IV; knjEx260[odr-7p::nhr-76(cDNA) + ccRFP + rps-0p::HygR]*	AWA-specific *nhr-76* over expression with the *odr-7* promoter in the *nhr-76(knj51)* background (Line2)	This study, Injection
NUJ788	*nhr-76(knj51); knjEx262[odr-7p::nhr-76(cDNA) + ccRFP + rps-0p::HygR]*	AWA-specific *nhr-76* over expression with the *odr-7* promoter in the *nhr-76(knj51)* background (Line3)	This study, Injection
NUJ789	*nhr-76(knj39)IV; knjEx234[ccRFP + rps-0p::HygR]*	Control for the co-injection markers (Hygromicine resistantce and Coelomocyte RFP) (Line1)	This study, Injection
NUJ792	*nhr-76(knj39)IV; knjEx235[ccRFP + rps-0p::HygR]*	Control for the co-injection markers (Hygromicine resistantce and Coelomocyte RFP) (Line2)	This study, Injection
NUJ567	*"nhr-76(knj39)IV; knjEx226[nhr-76p::nhr-76 + ccRFP + rps-0p::HygR]"*	Genomic fragment rescue of *nhr-76* (Line1)	This study, Injection
NUJ568	*"nhr-76(knj39)IV; knjEx227[nhr-76p::nhr-76 + ccRFP + rps-0p::HygR]"*	Genomic fragment rescue of *nhr-76* (Line2)	This study, Injection
NUJ713	*knjEx250[odr-10p::nhr-76(cDNA) + ccRFP + rps-0p::HygR]*□ □	AWA-specific *nhr-76* over expression with the *odr-10* promoter	This study, Injection

Table S2 Oligonucleotides.

Table S2 Primers and crRNAs

Primer	Gene	Sequence (5' to 3')	Note
KN1170	*cdc-42*	CTGCTGGACAGGAAGATTACG	For qPCR (as a reference)
KN1171	*cdc-42*	CTCGGACATTCTCGAATGAAG	For qPCR (as a reference)
KN1251	*dpy-10*	GCUACCAUAGGCACCACGAGGUUUUAGAGCUAUGCU	crRNA for Co-CRISPR strategy
KN1624	*odr-10*	TTAGTACATTGGTGACAGCCGC	For qPCR
KN1625	*odr-10*	TTGGAATCGGCGCCAGACGG	For qPCR
KN1699	*odr-7*	CAAGCCGCGATGGAACTGGG	For qPCR
KN1700	*odr-7*	GTGCTGCACATTTTCTGGAAGC	For qPCR
KN1972	*nhr-76*	CUGACAUGUUCUGAGAACUCGUUUUAGAGCUAUGCU	crRNA for LBD deletion
KN1973	*nhr-76*	UUGAAACAACUAAAUGAAGUGUUUUAGAGCUAUGCU	crRNA for LBD deletion
KN2074	*nhr-76*	ATGGAGGTGCTCGGGAAGC	For electro phoresis (mRNA of *nhr-76*)
KN2075	*nhr-76*	TTACGTGAACGCGAGATCATCAA	For electro phoresis (mRNA of *nhr-76*)
KN2076	*nhr-76*	GTCGGCTATCTCTGGTGACG	For generating genomic fragment of *nhr-76*
KN2077	*nhr-76*	GCCCACTACTTAGACCACGAG	For generating genomic fragment of *nhr-76*
KN2082	*nhr-76*	CUGCCAGUUGGGGAAGUAUGGUUUUAGAGCUAUGCU	crRNA for CRISPR KO
KN2083	*nhr-76*	GCUGAUGGAAGGCACGAGAAGUUUUAGAGCUAUGCU	crRNA for CRISPR KO
KN2087	*nhr-76*	TTTGTACAAAAAAGCAGGCTCCGAATTCATGGAGGTGCTCGGGAAGCA[illegible]	For generating *nhr-76* cDNA
KN2088	*nhr-76*	TTGTACAAGAAAGCTGGGTCGAATTCTTACGTGAACGCGAGATCATCA[illegible]	For generating *nhr-76* cDNA
KN2303	*nhr-76*	TGCTGTGTCATTGGCCATGC	For sequencing and PCR
KN2317	*nhr-76*	TGGACAGCTCACCGGGTA	For qPCR
KN2331	*nhr-76*	GTGGAGATGGCTTCATATAGGCG	For qPCR
KN2332	*nhr-76*	CTCTGTGGAGCGACGTCG	For PCR
KN2570	*nhr-76*	CGAGTCGTAACGCCGTAGTG	For PCR
KN2572	*nhr-76*	CCACATGAGGCTGGTCTTCTCG	For qPCR
KN2573	*nhr-76*	TCACGGTGAACTGCCATCG	For qPCR
KN2574	*nhr-76*	CCGATCAAATTGTCGAGCATG	For PCR
KN2576	*nhr-76*	ACTCGATCAAGCCACGGTCC	For PCR
KN2577	*nhr-76*	GGTCCTACACGGATTTGAGT	For PCR

Table S3 Plasmids.

Table S3 Plasmids

Plasmid	Description	Related strains	Note
pCZGY66	*rgef-1p::GTW-unc-54 3'UTR*		Gift from Yishi Jin
pKEN838	*ges-1p::GTW-3'UTR(unc-54)*		
pKEN927	*odr-7p::GTW-3'UTR(unc-54)*		
pKEN928	*odr-10p::GTW-3'UTR(unc-54)*		
pKEN1071	*nhr-76(cDNA)* pCR8 Backborne		
pKEN1072	*rgef-1p::nhr-76(cDNA)*	NUJ605, NUJ612	pKEN1071 + pCZGY66
pKEN1073	*ges-1p::nhr-76(cDNA)*	NUJ607, NUJ608	pKEN1071 + pKEN838
pKEN1074	*odr-10p::nhr-76(cDNA)*	NUJ609, NUJ611, NUJ713	pKEN1071 + pKEN927
pKEN1075	*odr-7p::nhr-76(cDNA)*	NUJ633, NUJ634, NUJ635, NUJ788, NUJ790, NUJ791	pKEN1071 + pKEN928
pKEN939	*odr-10p::tagRFP*	NUJ636	
pRF4	*rol-6(su1006)*	NUJ636	
pUC19		NUJ587, NUJ588, NUJ576, NUJ590, NUJ605, NUJ612, NUJ607, NUJ608, NUJ609, NUJ611, NUJ627, NUJ628, NUJ629, NUJ633, NUJ634, NUJ635, NUJ636, NUJ567, NUJ568, NUJ713, NUJ788, NUJ789, NUJ790, NUJ791, NUJ792	
pKEN954	*rps-0shp::HygR*	NUJ587, NUJ588, NUJ576, NUJ590, NUJ605, NUJ612, NUJ607, NUJ608, NUJ609, NUJ611, NUJ627, NUJ628, NUJ629, NUJ633, NUJ634, NUJ635, NUJ567, NUJ568, NUJ713, NUJ788, NUJ789, NUJ790, NUJ791, NUJ792	
pKEN281	coelomocyte RFP (ccRFP)	NUJ587, NUJ588, NUJ576, NUJ590, NUJ605, NUJ612, NUJ607, NUJ608, NUJ609, NUJ611, NUJ627, NUJ628, NUJ629, NUJ633, NUJ634, NUJ635, NUJ567, NUJ568, NUJ713, NUJ788, NUJ789, NUJ790, NUJ791, NUJ792	
